# A specialist herbivore pest adaptation to xenobiotics through up-regulation of multiple Cytochrome P450s

**DOI:** 10.1038/srep20421

**Published:** 2016-02-10

**Authors:** Fang Zhu, Timothy W. Moural, David R. Nelson, Subba R. Palli

**Affiliations:** 1 Department of Entomology, University of Kentucky, Lexington, KY 40546, USA; 2 Department of Entomology, Washington State University, Pullman, WA 99164, USA; 3 Department of Chemistry, Washington State University, Pullman 99164, USA; 4 Department of Microbiology, Immunology and Biochemistry, University of Tennessee, Memphis, TN 38163, USA

## Abstract

The adaptation of herbivorous insects to their host plants is hypothesized to be intimately associated with their ubiquitous development of resistance to synthetic pesticides. However, not much is known about the mechanisms underlying the relationship between detoxification of plant toxins and synthetic pesticides. To address this knowledge gap, we used specialist pest Colorado potato beetle (CPB) and its host plant, potato, as a model system. Next-generation sequencing (454 pyrosequencing) was performed to reveal the CPB transcriptome. Differential expression patterns of cytochrome P450 complement (CYPome) were analyzed between the susceptible (S) and imidacloprid resistant (R) beetles. We also evaluated the global transcriptome repertoire of CPB CYPome in response to the challenge by potato leaf allelochemicals and imidacloprid. The results showed that more than half (51.2%) of the CBP cytochrome P450 monooxygenases (P450s) that are up-regulated in the R strain are also induced by both host plant toxins and pesticide in a tissue-specific manner. These data suggest that xenobiotic adaptation in this specialist herbivore is through up-regulation of multiple P450s that are potentially involved in detoxifying both pesticide and plant allelochemicals.

The adaptation of herbivorous arthropod pests to their host plants is hypothesized to be intimately connected with the ubiquitous development of pesticides resistance in agricultural environment^1^,^2^,^3^. However, the molecular mechanisms underlying these connections are still not well known. Many synthetic pesticides resemble or are even derived from plant allelochemicals (e.g. pyrethroids, neonicotinoids), hence, it is likely that the strategies utilized by herbivore pests to surmount a defense to plant toxins and pesticides are very similar^3^,^4^,^5^,^6^. These strategies include avoidance behavior, a reduction in penetration, an increase in excretion, sequestration and detoxification, and target-site insensitivity^7^,^8^. In different insect systems, these strategies independently or simultaneously contribute to adaptation to plant toxins and/or pesticides. Among these, metabolic detoxification through cytochrome P450s, glutathione S-transferases (GSTs,) uridine diphosphate-glycosyl transferases (UGTs) and carboxyl-esterases (COEs) have been considered as the most dominant cross-resistance mechanisms linking host plant toxins adaptation with pesticide resistance^7^,^9^,^10^,^11^.

Herbivorous arthropods are conventionally classified into two categories, generalists (polyphagous) and specialists (monophagous and oligophagous)^12^. The generalists feed on more than one plant family, whereas the specialists only consume one or a few closely related plant taxa within one botanical family. Since generalists encounter a wide range of plant toxins in their diet, it is often assumed that generalists likely possess a greater capacity to detoxify plant allelochemicals than specialists^13^, which in turn partially impacts the development of broad pesticide resistance^3^. While specialist herbivores tend to have highly efficient detoxification mechanisms fine-tuning their adaptation to a limited spectrum of host plants defense^13^. A recent study on a generalist, two-spotted spider mite, revealed that the transcriptional profiles of tomato-adapted mites resembled those of multi-pesticide-resistant populations. The adaptation to tomato increased the resistance to a few unrelated pesticide classes^3^. Additionally, a study on the generalist cotton bollworm showed that plant allelochemicals-induced P450 genes participated in tolerance to a pyrethroid insecticide^14^. However, this specialist-generalist paradigm classified based on the host range does not precisely reflect the potential of an herbivore pest to develop pesticide resistance (Table 1). More than 90% of all herbivore insects feed on plants in less than three families^15^. And many specialists are agriculturally important pests with a remarkable ability to develop resistance to a wide range of pesticides^13^,^16^,^17^ (Table 1). To date, there is no study on genome-scale transcriptional responses of specialist herbivores to pesticides and their host plants.

Here, we used Colorado potato beetle (CPB), *Leptinotarsa decemlineata*, as a unique archetype to reveal how a specialist herbivore pest copes with host plant defense and synthetic pesticides. The host plants of CPB are limited to nightshade plants (the family Solanaceae), including potatoes a globally important food crop containing extremely toxic glycoalkaloids (GAs) in many parts of the potato plants^17^. Both CPB larva and adults consistently consume potato foliage during their development. In parallel with CPB adaptation to host plant toxins, CPB has an exceedingly high record of developing pesticide resistance. From the middle of the last century, CPB has developed resistance to more than 50 pesticides within all five major classes of synthetic pesticides^17^ (Table 1). As an ideal model, CPB serves as a representative in the most species-rich eukaryotic order, Coleoptera, containing about one-third of the herbivore insect species. The functional genomics tool, RNA interference (RNAi)-mediated gene silencing, works systemically in CPB^18^,^19^ and therefore could facilitate deciphering molecular mechanisms underlying the connection between detoxification of plant toxins and synthetic pesticides.

In this study, we focused on cytochrome P450 complement (CYPome) because previous biochemical and physiological studies showed that cytochrome P450-mediated detoxification is the most common mechanism involved in CPB neonicotinoid resistance^17^,^20^. Due to their large numbers and broad substrate spectrum, cytochrome P450s are among the most vital groups of enzymes contributing to both host plant adaptation and development of pesticide resistance^3^,^14^. In the present study, we first conducted next-generation sequencing (454 pyrosequencing) to determine CPB transcriptome from the susceptible (S) and imidacloprid resistant (R) beetles. Then we compared the transcriptional patterns of CPB CYPome between S and R beetles with quantitative real time PCR (qRT-PCR). Finally, the global transcriptional profiles of CPB CYPome from various tissues responding to pesticide and plant allelochemicals were investigated.

## Results

### CPB transcriptome analysis

Roche 454 pyrosequencing of CPB RNA resulted in a total of 567,940 reads constituting 112,752,502 bases. The high quality reads were combined with 9,302 publicly available CPB sequences downloaded from the NCBI website and were submitted to Roche *de novo* Assembler program (Newbler). These sequences were assembled into 99,491 ESTs including 13,521 contigs (Fig. S1A) and 85,970 singletons (Fig. S1B). The average length of contigs was 560 bp (Fig. S1A). NCBI blastx database searches at e^-3 identified homologues for contigs and singletons. The distribution of blast hits among various species exhibited that the maximum number of blast hits were with the sequences of *Tribolium castaneum*. 81.4% of sequences shared the greatest sequence similarity with *T. castaneum,* followed by 1.4% for *Apis mellifera* and 1.3% for *Nasonia vitripennis* (Fig. S2). The Gene Ontology (GO) annotation grouped all contigs into biological processes, and about 21% of genes were classified into the metabolic process (Fig. S3A). The other GO categories, cellular component and molecular function (ontology level 2), are shown in Fig. S3B and C, respectively.

### CPB CYPome discovery

**Figure 1:**
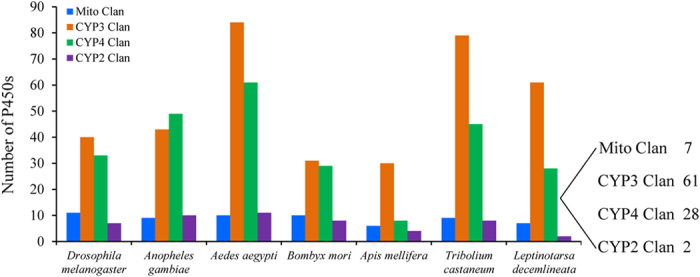
The column chart showing P450 gene numbers discovered in several insect species. The blue, orange, green, and purple columns stand for Mito clan, CYP3 clan, CYP4 clan and CYP2 clan, respectively.

In the current study, 98 cytochrome P450s were annotated from 99,491 ESTs and named by the P450 nomenclature committee (Tables S1 and S2). Among these 98 P450 genes, two (CYP18 and 305 families) derived from CYP2 clan, seven (CYP12, 49, 301, 314 families) belong to Mito clan, 61 (CYP6, 9, 345, 347, 413 families) derived from CYP3 clan and 28 (CYP4, 349, 350, 411, 421) belong to CYP4 clan (Table S1, Fig. 1). *L. decemlineata* has largely expanded CYP3 (20 subfamilies, 61 individual genes) and CYP4 (7 subfamilies, 28 individual genes) clans, especially the families 6 (23 genes), 9 (32 genes), and 4 (21 genes), which consist of 76 genes of all CPB P450s identified (Table S1). A number of P450 genes in these three families are known to be involved in detoxification of plant allelochemicals as well as resistance to pesticides through their constitutive overexpression and/or inducible expression in R strains^2^,^11^. Therefore, the rest of our studies focused on these 76 P450 genes belonging to the three families.

### Differential expression of CPB P450s in S and R beetles

**Figure 2:**
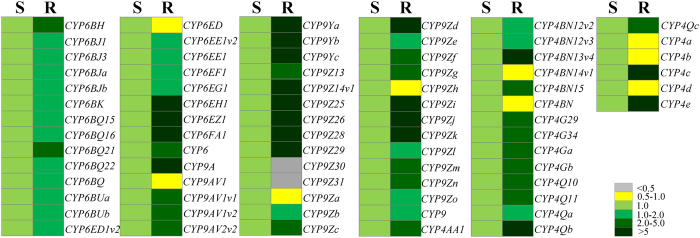
A large number of CPB P450 genes are overexpressed in the R strain. The relative expression ratios of 76 CPB P450s in imidacloprid Resistant (**R**) strains compared with those in the Susceptible (**S**) strain by qRT-PCR are shown. The rectangles are colored on the basis of mean (n = 6) relative mRNA levels in S and R strains. The levels of relative expression are illustrated by a six- category color scale as shown in the legend. The relative expression ratios between R and S strain and p-values are listed in Supplementary Material: Table S4.

**Figure 3:**
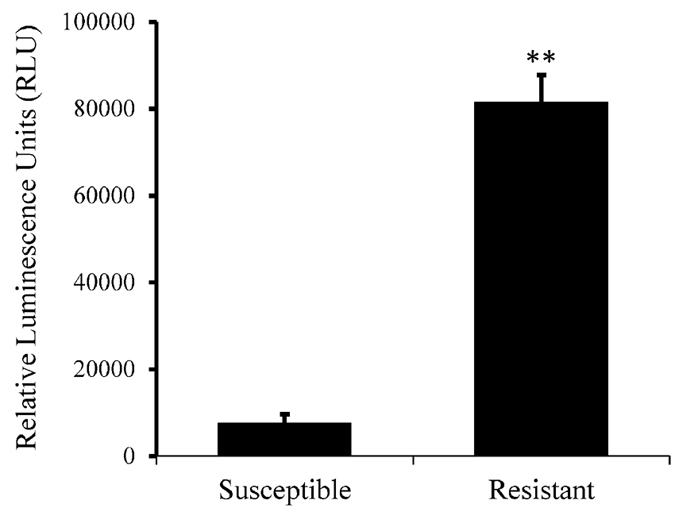
Cytochrome P450 activity in the gut of S and R female adults. The gut (foregut and midgut) was dissected from one week-old female CPB adult under ice-cold 1× PBS solution. Luminescent P450 activity assays were performed using commercially available (Promega) P450-Glo substrates Luciferin-Be, Luciferin-H, or Luciferin Me. The data shown are the mean + SEM (n=3). Statistical significance of the gene expression between two samples was calculated using Student’s t test. **p-value < 0.01.

To identify the P450s that might be involved in the imidacloprid resistance in the R strain, we first examined the relative expression of 76 P450s belonging to CYP4, 6, and 9 families between S and R strains (whole body of new emerged female adults) in comparison with the expression of the most stable housekeeping genes *RPL4* and *Ef1α* (Table S3). There are 41 P450s (54% of 76 P450s) showing more than a 2-fold up-regulation (*p* value < 0.05) in the R strain compared to their expression in the S strain (Fig. 2; Table S4). Only two P450s (2.6% of 76 P450s) exhibited significant down-regulation (< 0.5 fold, *p* value < 0.05) in the R strain compared to their expression in the S strain (Fig. 2, Table S4). These 41 overexpressed P450s include 22 CYP9, 13 CYP4, and 6 CYP6 genes, suggesting multiple P450s may contribute to imidacloprid resistance in the R strain. Simultaneously, to decipher the difference in the P450 enzyme activities between S and R strains, we assayed the P450 levels in the gut of 1 week old S and R female beetles after removal of their lumen contents. As shown in Fig. 3, the total P450 activity in the R strain is 10.96-fold higher (*p* value < 0.0001) than that in the S strain. Taken together, these data suggest the enhanced expression of P450 genes results in higher level of P450 enzymes in the R strain which may contribute the resistance to imidacloprid.

### Tissue specific induction of CPB P450s in R beetles

**Figure 4:**
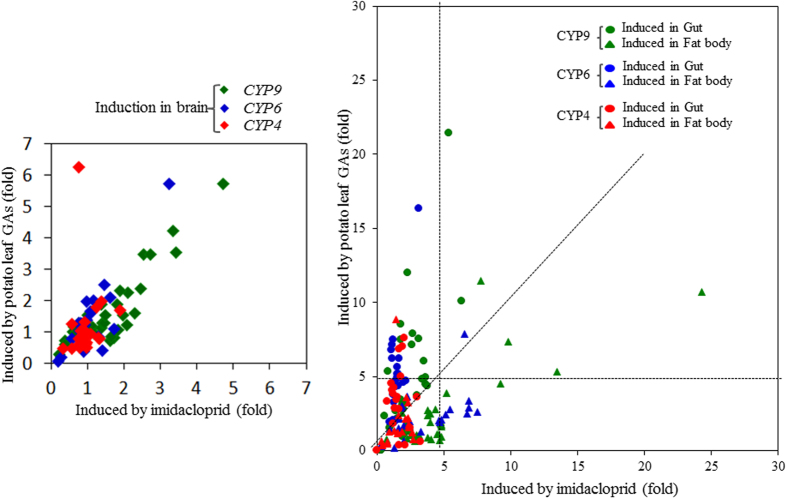
Induction of CPB P450s by potato leaf GAs and imidacloprid in the R strain. The scatter plots show the induction of 76 P450 genes by potato leaf GAs (Y axis) and imidacloprid (X axis) in the (**A**) head (with antennae) and (**B**) gut (foregut and midgut) (●) and fat body (▲) of female CPB R adults. The green, blue and red colors represent genes from CYP9, CYP6 and CYP4 families, respectively. The fold induction was calculated based on relative mRNA levels of P450 genes in one tissue dissected from beetles fed on potato leaf GAs in comparison with that in the control beetles fed on artificial food or induced by imidacloprid in comparison with that in the control beetles treated with ethanol only. The relative expression ratios between induced and uninduced and p-values are listed in Table S5.

In many cases, comparison of only two strains (S and R) leads to differences associated with variances in the genetic background. For accuracy, we then screened CPB P450s induced by potato leaf GAs and imidacloprid in the R strain which may contribute the resistance to imidacloprid and adaption to host plant toxins. The relative expression of 76 P450s induced by potato leaf GAs (24 hours) and imidacloprid (6 hours) in three key xenobiotic metabolism tissues: head, fat body, and gut of female R beetles were examined. In the head, only 10 out of 76 P450s were induced by imidacloprid, and 13 P450s were induced by potato leaf GAs by >  2-fold with a *p* value < 0.05 (Fig. 4A; Table S5). However, in the fat body, 51 out of 76 P450s were induced by imidacloprid, and 29 were induced by potato leaf GAs by >  2-fold with a *p* value < 0.05 (Fig. 4B; Table S5). In the gut, 24 and 56 P450s were induced by imidacloprid and potato leaf GAs, respectively (Fig. 4B; Table S5). These data suggest that host plant toxins and pesticides impact more on the expression of P450s in the fat body and gut than in the head. It is interesting that 51 P450 genes were induced by imidacloprid in the fat body when compared to 24 in the gut. In contrast, 56 P450 genes were induced by potato leaf GAs in the gut compared to 29 in the fat body (Fig. 4B; Table S5). Moreover, in the gut, most genes in CYP9 and CYP6 were induced to higher levels by potato leaf GAs than by imidacloprid. The same genes were induced to higher levels by imidacloprid than by potato leaf GAs in the fat body. The CYP4 P450s were induced by potato leaf GAs to higher levels than the induction by imidacloprid in both gut and fat body. However, a significantly smaller number of CYP4 genes were induced (> 5 fold) than CYP6 and CYP9 genes (Fig. 4B; Table S5).

### Multiple P450s up-regulated by host plant toxins and pesticide

**Figure 5:**
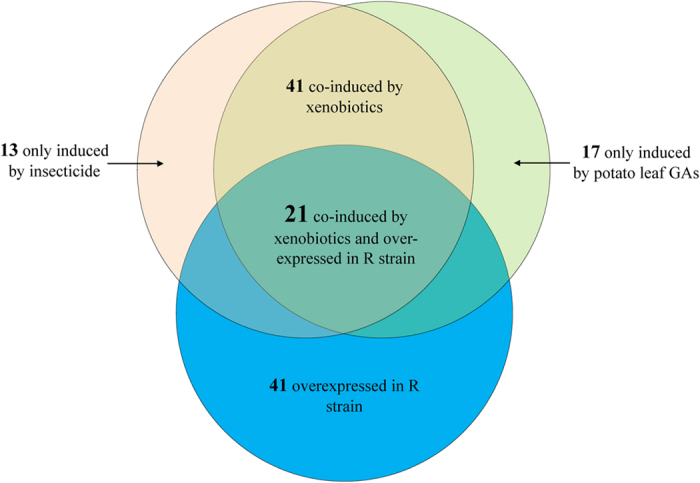
Venn diagram representing the overlapping of induction and overexpression of CPB P450s. P450s induced by potato leaf GAs in either gut or fat body of female CPB R adults are shown in green circle. P450s induced by imidacloprid are shown in pink circle (induction ratio > 2-fold; *p* < 0.05; details are listed in Table S5). The blue circle exhibits P450s that are overexpressed in the R strain than the S strain by > 2-fold and a *p* value < 0.05 (Table S4).

Figure 5 shows the overlap in induction and overexpression of CPB P450s. Forty-one out of 76 P450s were induced by both potato leaf GAs and imidacloprid by >  2 fold with a *p* value < 0.05 (Fig. 5 and Table S5). Thirteen P450s were specially induced by imidacloprid and 17 P450s were induced by potato leaf GAs alone. Twenty-one of these 41 P450s (51.2%) overexpressed in the R strain were also induced by both potato leaf GAs and imidacloprid (Figs 2 and 5). In insects, constitutive overexpression and/or induction in pesticide resistant strains are hallmarks of P450s associated with pesticide detoxification^11^,^21^. Many insect P450s that are involved in host plant toxins adaptation are shown to be induced by plant allelochemicals^2^. The multiple P450s that are up-regulated by both host plant toxins and pesticide may play roles in both host plant adaptation and pesticide resistance.

### Phylogenetic relationship of xenobiotic induced CPB P450s with other insect P450s

**Figure 6:**
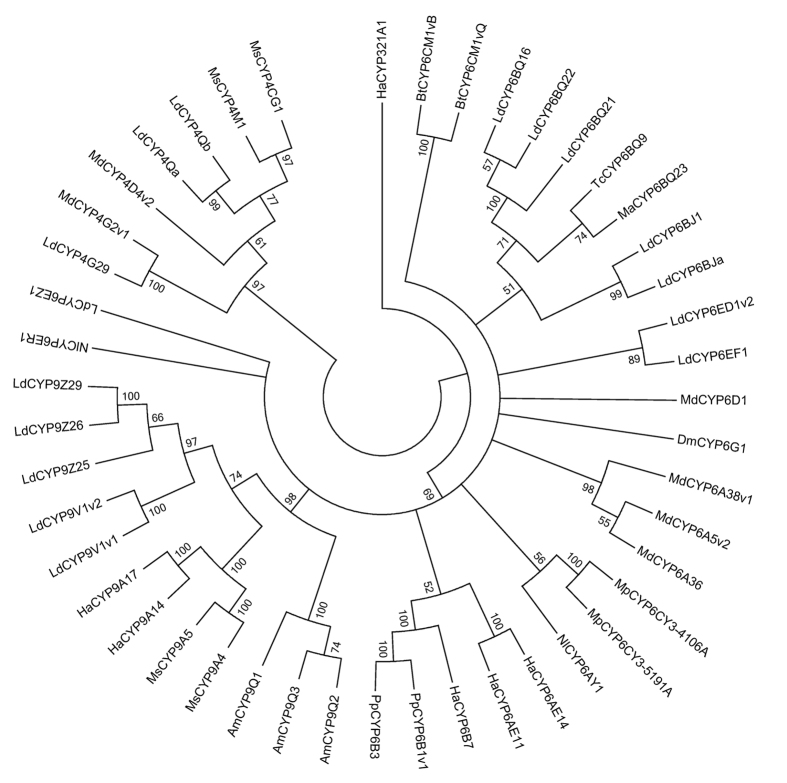
Phylogeny of P450 genes involved in xenobiotics adaptation among insect species. Am, *Apis mellifera*; Bt, *Bemisia tabaci*; Dm, *Drosophila melanogaster*; Ld, *Leptinotarsa decemlineata*; Ha, *Helicoverpa armigera*; Ma, *Meligethes aeneus*; Md, *Musca domestica*; Mp, *Myzus persicae*; Ms, *Manduca sexta*; Nl, *Nilaparvata lugens*; Pp, *Papilio polyxenes*; Tc, *Tribolium castaneum*. The phylogenetic consensus tree was generated by MEGA v6.06 according to the amino acid sequences. The branch support was given by posterior probabilities. The condensed tree was created with cut-off value of 50%.

Phylogenetic analysis was conducted based on the amino acid sequences of 46 insect P450s (Fig. 6). Sixteen representative CPB P450s induced by both potato leaf GAs and imidacloprid were selected based on the MUSCLE alignment. Thirty P450s from other insects were chosen based on their association with pesticide resistance and/or host plant adaptation (Table S6). The neighbor-joining tree grouped 3 CPB *CYP6BQ*s with *Meligethes aeneus CYP6BQ23* and *T. castaneum CYP6BQ9* with 71% bootstrap support (Fig. 6). Five CBP *CYP9*s were clustered with *CYP9A14* and *CYP9A17* in *Helicoverpa armigera* and *CYP9A4* and *CYP9A5* in *Manduca sexta* (Fig. 6). *CYP4Qa* and *CYP4Qb* were grouped with *CYP4CG1* and *CYP4M1* in *M. Sexta. CYP4G29* originated from the same evolutionary root with *CYP4G2v1* in *Musca domestica* (Fig. 6).

## Discussion

In the current study, we used the specialist herbivore pest CPB to evaluate the global transcriptome repertoire of CPB P450s in response to host plant allelochemicals and pesticide. The higher P450 activities in the gut of R beetles than the S beetles (Fig. 3) provides evidence that these P450s play a critical role in metabolizing the synthetic pesticide which the R beetles resist to. The differential expression and induction experiments suggest that multiple P450 genes may contribute to the detoxification of host plant allelochemicals and topically applied pesticide (Figs 2, 4 and 5).

Here, we revealed genome-scale transcriptional profiling of P450s from a specialist herbivore in response to pesticide and its host plant allelochemicals. Our study identified 41 P450s showing constitutive overexpression in the R strain (Figs 2 and 5). Among these P450s, 21 of them were also induced by both host plant toxins and pesticide (Fig. 5). These results strongly suggest xenobiotic adaptation in this specialist herbivore is possible through up-regulation of multiple P450s. Schuler predicted that if the acquisition of pesticide resistance is a consequence of particular P450s that detoxify pesticides in addition to host plant allelochemicals, the costs and limitation for this resistance would be little^4^. As with constitutive overexpression, induction causes an activity enhancement of the detoxification system that leads to an increase in metabolic defenses^22^. However, the metabolism of toxins by insects requires a significant amount of energy^23^. Rather than maintaining a high level of expression all the time as in constitutive overexpression, induction increases the amount of enzymes produced only when a chemical stimulus occurs, representing an adaptive plasticity or trade-off between conserving energy and survival in a chemically unfriendly environment^4^,^7^,^14^,^21^,^22^. Therefore, induced genes are more likely to become involved in resistance than non-induced genes. This type of cross-resistance between allelochemicals and pesticides metabolism that allow insects to acquire robust pesticide resistance by ingestion of food constituents would have little effect on insect fitness. To decipher which specific gene(s) facilitate the cross-resistance between allelochemicals and pesticides metabolism, further experiments are in progress.

Insect P450s are known to be expressed in a tissue-specific pattern. For example, most of *Drosophila* P450s are expressed in more than one epithelial tissues^24^. Previous metabolic studies related to pesticides and plant allelochemicals focused on midgut, fat body, head, and Malpighian tubules^24^,^25^,^26^. Different P450 genes exhibited diverse induction patterns in response to chemicals. For instance, *Papilio polyxenes* CYP6B1 and CYP6B3 in the midgut contributed to the metabolism of toxic furanocoumarins from its host plant. *CYP6B1* was induced by xanthotoxin in all tissues tested, while *CYP6B3* was induced only in the fat body^27^. Comparisons between the promoters of these two P450s indicated that these two genes contained variations in their chemical-response elements. In our study, the investigation of CPB P450s induction by imidacloprid and potato GAs was conducted in three major xenobiotic metabolism tissues, head, fat body and gut (Fig. 4). The tissue specific approach prevents omission of important candidate genes because some expression changes likely disappear in the measurement of gene expression of pooled insect tissues, especially for the tissue specifically expressed genes. As shown in Fig. 4A, few P450 genes were induced by either potato leaf GAs or imidacloprid in the head. Whereas, more than half CPB P450s were induced by imidacloprid in the fat body or induced by potato leaf GAs in the gut (Fig. 4B). The diverse P450s tissue specific expression response to xenobiotic challenges may vary partially due to distinct exposure methods. For example, ingestion of potato leaf GAs induced more P450s in the gut than in the fat body. Conversely, topical application of imidacloprid induced more P450s in the fat body than in the gut (Fig. 4B). Although both CYP3 and CYP4 clans are largely expanded in CPB (Fig. 1), more CYP3 clan P450s than CYP4 clan P450s were induced by both potato leaf GAs and imidacloprid (Fig. 4B), indicating the vital roles of CYP3 clan P450s in the xenobiotic detoxification in the R beetles.

Previous research suggested that pesticide resistance can be caused by one or more than one P450s that are constitutively overexpressed and/or induced by pesticides in the R strain. For example, the constitutive overexpression of *CYP6D1*, a house fly P450 was responsible for pyrethroid resistance in the LPR strain^28^. The overexpression of a single cytochrome P450, *CYP6g1*, conferred DDT resistance in DDT resistant strains of *Drosophila melanogaster*^29^. This protein was also directly linked to the enhanced metabolism of imidacloprid *in vivo*^30^. *CYP6BQ9*, a red flour beetle P450 contributed to the deltamethrin resistance of the *T. castaneum* QTC279 strain through both constitutive overexpression and induction^26^,^31^. The overexpression of a P450, *CYP6BQ23*, was the primary mechanism conferring pyrethroid resistance in pollen beetle populations in Europe^32^. Our phylogenetic analysis clustered several CPB *CYP6* genes with *CYP6BQ9* and *CYP6BQ23* (Fig. 6), suggesting the potential functions of these P450s in detoxification of pesticides. In the whitefly, *Bemisia tabaci*, the constitutive overexpression of a single P450 *CYP6CM1* was shown to be associated with imidacloprid resistance in both B and Q biotypes^33^. In the peach aphid, *Myzus persicae*, the constitutive overexpression of a P450 *CYP6CY3* contributes to the neonicotinoid resistance^34^. The overexpression of two P450s, *CYP6ER1* and *CYP6AY1*, are closely linked with the imidacloprid resistance in the brown planthopper, *Nilaparvata lugens*^35^,^36^,^37^. The up-regulation through induction and/or constitutive overexpression of *CYP4D4v2, CYP4G2v1, CYP6A5v2, CYP6A38v1*, and *CYP6A36* was found to be associated with permethrin resistance of ALHF house flies^21^,^38^,^39^,^40^. However, many other P450s have been reported to be responsible for metabolism of plant allelochemicals through induction. For example, CYP6B enzymes in insects belonging to order Papilionidae were induced by toxic furanocoumarins and these enzymes converted furanocoumarins to nontoxic compounds. CYP6B enzymes are considered as one of the key factors that allow caterpillars adapt to the poisonous host plant^41^,^42^. Some CYP4 and CYP9 P450s in *M. sexta* are induced by various classes of host plant allelochemicals^25^,^43^,^44^. Several deltamethrin and gossypol induced P450s that participated in both deltamethrin and gossypol tolerance in *H. armigera* have also been identified^14^,^45^. In our study, five CBP CYP9 P450s displayed close evolutionary relationship with these P450s including *HaCYP9A14, HaCYP9A17, MsCYP9A4* and *MsCYP9A5* (Fig. 6).

In conclusion, the current study provides a cohort of candidate P450s that are potentially involved in metabolizing host plant toxins and pesticides. It sheds new light on understanding herbivorous pest adaptation to their host plants along with their ubiquitous development of resistance to synthetic pesticides. Further functional studies are under way to determine which specific gene or genes are responsible for the host plant adaptation and pesticide resistance.

## Methods

### CPB strains

The susceptible CPB strain (S) was kindly provided by Dr. Don Weber of USDA-ARS, originally supplied by the New Jersey Department of Agriculture. The imidacloprid resistant CPB strain (R) was collected from Long Island, NY. The resistance ratio of R compared with the S for imidacloprid (technical grade, 99.5% purity; Chem Services, West Chester, PA) was about 30 times in topical application bioassay studies. Beetles were reared on Red Norland potato plants at 25 ± 5 °C under a light:dark regimen of 16:8 h in BugDorm insect cages (MegaView Science Co., Ltd.). Each strain was maintained in several insect cages in the greenhouse at University of Kentucky. New potted potato plants were provided twice each week. Egg masses were collected daily and maintained in a petri dish with water soaked filter paper and kept in an incubator (26 ±1 °C, RH of 70%, L:D 16:8). The young larvae were set to feed on fresh potato leaves after they emerged and then transferred back to their colony in the greenhouse during the 2^nd^ instar stage.

### RNA isolation, cDNA library construction, and 454 sequencing

To reveal the transcriptome of CPB, we performed 454 GS FLX pyrosequencing following previously published protocols^46^. We collected three larvae, pupae, male and female adults, respectively, as well as 30 eggs at 24, 48, 72, 96 and 120 h after oviposition, from both S and R beetles for RNA extraction. We isolated total RNA from these samples using TRIZOL reagent (Invitrogen) following manufacturer’s protocol and the RNA from S and R strains were combined in equal proportions. The quality of total RNA was checked by gel electrophoresis and spectrometry analyses. Poly A^+^ RNA was isolated using Poly(A) Track mRNA isolation kit (Promega). The Poly A^+^ RNA was used to synthesize cDNA using SMART™ system (Clontech Laboratories) following manufacturers protocols except replacing oligo-dT primer with custom synthesized random primer. Stretches of Ts present in oligo-dT primed cDNAs could cause problems during pyrosequencing. About 10 *μ*g of cDNA was used for 454 pyrosequencing with Genome Sequencer FLX system (Roche-applied-science) available at Advanced Genomics Technology Center of University of Kentucky (http://www.uky.edu/Centers/AGTC/). cDNA was sheared into 300–800 bp fragments and adapter sequences were ligated following manufacturer’s methods. The adaptors were used in purification, amplification and sequencing steps.

### Bioinformatics

Emulsion PCR followed by sequencing was performed following manufacturer’s suggested methods. Initial quality filtering and Shotgun assembly of sequences were performed using GS De Novo Assembler (Newbler; Roche) provided by the instrument. We also used commercially available software (Seqman Pro; http://www.dnastar.com/products/seqmanpro.php) for assembly. All assembled ESTs were screened for vector sequence contamination using Seqman Pro’s vector screening software. The assembled contigs were aligned against the UniVec Core vector database from NCBI (ftp://ftp.ncbi.nih.gov/pub/UniVec) and all the contaminating vector sequences were removed. NCBI blastx database searches identified homologous for contigs and singletons were set up with an e-value cut-off at 10^−3^. All sequences were searched in databases such as complete Uniprot annotated protein database (http://www.expasy.uniprot.org/database/downloads.html), *Bombyx mori* protein set (http://silkworm.genomics.org.cn), *D. melanogaster* unigenes (Flybase: http://flybase.bio.indiana.edu) and *T. castaneum* database (http://beetlebase.org). Functional annotation was performed using Blast2GO software (http://www.blast2go.com/b2ghome), assigning Gene Ontology (GO) terms, and the metabolic pathways were predicted in Kyoto Encyclopedia of Genes and Genomes (KEGG). The GOSlim available in Blast2GO was used to group contigs into biological process, cellular component, and molecular function at ontology level 2, indicating general functional categories.

### Quantitative real time PCR (qRT-PCR) and reference gene selection

qRT-PCR was performed in Applied Biosystems StepOnePlus^TM^ Real-Time PCR System (Life technologies^TM^, Carlsbad, CA). Total RNA was isolated from three (whole body) or six (tissues) female R or S beetles using the TRI reagent (Molecular Research Center Inc., Cincinnati, OH) and the RNA was treated with DNase I (Ambion Inc., Austin, TX). cDNA was synthesized using iScript cDNA synthesis kit (Bio-Rad Laboratories, Hercules, CA) and DNase I treated total RNA was used as a template. Each qRT-PCR reaction (10  *μ*L final volume) contained 5  *μ*L FastStart SYBR Green Master (Roche Diagnostics, Indianapolis, IN), 1.0  *μ*L of cDNA, 3.6  *μ*L ddH_2_O, and 0.4  *μ*L each of forward and reverse gene specific primers (Table S7, stock 10 *μ*M). An initial incubation of 95 °C for 3 min, followed by 40 cycles of 95 °C for 10 s, 55 °C for 60 s settings were used. The qRT-PCR for each sample was conducted with two technique replicates and three biological replicates. The positive and negative (no-template) controls as well as internal controls were included in each plate. To evaluate the most stable reference genes among samples (S and R beetles, different tissues and development stages, control and induction by potato leaves or imidacloprid) for inter-run controls, five commonly used housekeeping genes, ribosomal protein L4 (*RPL4*), ribosomal protein L18 (*RP18*), elongation factor 1α (*Ef1α*), NADH dehydrogenase (*NADH*), heat shock protein 70 (*HSP70*) were analyzed with the *Bestkeeper* software package. We designed primers for reference genes according to EST sequences in NCBI database (Table S7). The most stable genes evaluated from the reference gene selection were chosen as reference genes for internal controls. Relative expression levels for target genes, in relation to the two most reliable reference genes were calculated by the 2^−∆∆CT^ method. Both the PCR efficiency and R^2^ (correlation coefficient) value were taken into consideration in estimating the relative quantities. PCR efficiencies between 95% and 105% and R^2^ value (correlation coefficient) > 0.99 for each gene were considered as qualified for further analysis.

### Luminescent P450 activity assay

The luminescent P450 activity assay was performed to evaluate if the P450 enzyme activities in the gut is consistent with the transcriptional expression of P450 genes in S and R beetles. The gut (foregut and midgut) was dissected from four one-week old female S or R CPB adults under ice-cold phosphate-balanced salt (PBS) solution, pH 7.2. After removal of gut contents, the gut was transferred to 100  *μ*L ice-cold homogenization buffer (HB: 0.1 M sodium phosphate, pH 7.5, containing 1 mM EDTA, 1 mM phenylmethylsulphonyl fluoride [PMSF], 0.1 mM dithiothreitol [DTT], and 1 mM 1-phenyl-2-thiourea [PTU]) and homogenized thoroughly with pestle. The concentration of total protein in the gut was measured by the Bradford dye binding assay. Luminescent P450 activity assays were performed in all-white 96-well plates (Thermo Fisher Scientific, Hudson, NH) using commercially available P450-Glo (Promega, Madison, WI) substrates Luciferin-Be, Luciferin-H, or Luciferin Me and equal amount of protein from guts dissected from S and R strains following the protocol described in Inceoglu *et al*. 2009^47^. This experiment was repeated three times.

### Induction by potato leaf GAs and imidacloprid

We used the starved (12–24 hours) newly emerged female CPB S or R adults to perform induction experiments. The control beetles for potato leaf GAs induction were fed on artificial diet (Colorado potato beetle diet, Bio-Serv, Frenchtown, NJ) for 48 hours. This artificial food consists of agar, casein, sucrose, fructose, salt, cholesterol, ascorbic acid, I-Inositol, fiber, locust bean gum, casein hydrolyzate, corn oil, choline dihydrogen citrate, and vitamin mix. It had been shown that the artificial food without plant toxins provided essential nutrition for the normal growth of CPB^48^. For potato leaf GAs treatment, the beetles were fed on artificial food for 24 hours and then fed potato leaves for 24 hours (based on our preliminary time course induction study). For imidacloprid treatment, the beetles were fed on an artificial diet for 48 hours. Then topical application of 1 *μ*L droplet of technical grade imidacloprid (1*μ*g; 99.5% purity; Chem Services, West Chester, PA) ethanol solution on the ventral surface of the abdomen were conducted during the final 6 hours of the 48 hours feeding period. We used 1*μ*g imidacloprid because our preliminary topical application bioassays showed LD_50 _= 1*μ*g/individual. Our initial bioassays suggested P450s exhibited an induction peak under the LD_50_ of imidacloprid, which was consistent with previous studies^21^,^49^. The beetles that fed on artificial diet for 48 hours and were treated with 1 *μ*L ethanol during the final 6 hours served as the control to imidacloprid induction. Every day 0.5 g fresh Red Norland potato leaves or artificial food were provided to each beetle. The food was replaced after 24 hours to avoid microorganism contamination. The beetles were kept in an incubator (26±1 °C, RH of 65 ± 5%, L:D 14:10). Six individuals were used for each treatment and control. After treatment, beetles were dissected into head (with antenna), gut (foregut and midgut), and fat body in ice-cold 1× PBS solution, frozen with liquid nitrogen, and then kept in −80 °C freezer until use. The induction experiment was repeated four times.

### Phylogenic tree construction

Thirty P450 amino acid sequences from other insect species (Table S6) were extracted from the National Center for Biotechnology Information (NCBI) (http://www.ncbi.nlm.nih.gov). These P450s and 16 representative of CPB P450s induced by xenobiotics were aligned with MUSCLE through MEGA v6.06 (http://www.megasoftware.net)^50^,^51^,^52^. A p-distance < 0.8 for the overall mean distance suggested the alignment was acceptable^52^. The phylogenic tree was estimated by the neighbor-joining algorithm as implemented by the MEGA program. Positions containing missing data and alignment gaps were eliminated in pairwise sequence comparisons. 2,000 bootstrap replicates were used.

### Statistical analysis

The statistical significance of the difference between control and treatments was calculated using a Student’s *t*-test (two-sample comparison). A value of *P* < 0.05 was considered statistically significant.

## Additional Information

**Accession codes:** RNA sequence reads have been submitted to NCBI under experiment #SRX1452307 and transcriptome #SRR2960806.

**How to cite this article**: Zhu, F. *et al*. A specialist herbivore pest adaptation to xenobiotics through up-regulation of Multiple Cytochrome P450s. *Sci. Rep.* **6**, 20421; doi: 10.1038/srep20421 (2016).

## Supplementary Material

Supplementary Information

## Figures and Tables

**Table 1:** Host plant range and pesticide resistance record of 10 agriculturally important arthropods.

Type	Name	Resistance to# of ActiveIngredients*	Host Plant Range	Reference
Generalist	Two-spotted spider mite,*Tetranychus urticae*	94	>1,100 species	Dermauw *et al*. 2013^3^
	Green peach aphid,*Myzus persicae*	76	>40 families	Silva *et al*. 2012a^53^; 2012b^54^
	Sweatpotato whitefly,*Bemisia tabaci*	55	>500 species	Rauch and Nauen 2003^55^; Yang *et al*. 2013^56^
	Cotton bollworm,*Helicoverpa armigera*	48	>120 species	Celorio-Mancera *et al*. 2012^57^; Tao *et al*. 2012^14^
	Beet armyworm,*Spodoptera exigua*	38	Wide host range	Bel *et al*. 2013^58^
Specialist	Diamondback moth,*Plutella xylostella*	92	Family Brassicaceae	You et al. 2013^16^; Xia *et al*. 2013^59^; Lei *et al*. 2014^60^
	Colorado potato beetle,*Leptinotarsa decemlineata*	55	Potato (*Solanum tuberosum* L.) and a few Solanaceae plants	Alyokhin *et al*. 2008^17^; Wan *et al*. 2013^61^; Kumar *et al*. 2014^62^
	Brown planthopper,*Nilaparvata lugens*	29	Rice (*Oryza sativa* L.) plants	Gorman *et al*. 2008^63^
	Western corn rootworm,*Diabrotica virgifera*	13	Corn (*Zea mays*) and a few others	Zhu *et al*. 2005^64^; Flagel *et al*. 2014^65^
	Tobacco hornworm,*Manduca sexta*	3	Tobacco (*Nicotiana tabacum*) and a few Solanaceae plants	Pauchet *et al*. 2010^44^; Feyereisen 2012^11^

^*^Arthropods resistant to Pesticides Database (ARPD): http://www.pesticideresistance.org (Accessed on December 30 2015).
