# Supplementary material for: A specialist herbivore pest adaptation to xenobiotics through up-regulation of multiple Cytochrome P450s
Source: Sci Rep. 2016 Feb 10;6:20421. doi: 10.1038/srep20421 (PMC4748221; doi:10.1038/srep20421)
Supplement: Supplementary Information [file srep20421-s1.pdf]

A specialist herbivore pest adaptation to xenobiotics through up-regulation of multiple Cytochrome P450s

Fang Zhu [1,2], Timothy W. Moural[3], David R. Nelson[4], and Subba R. Palli[1]

[1] Department of Entomology, University of Kentucky, Lexington, KY 40546, USA.

[2] Department of Entomology, Washington State University, Pullman, WA 99164, USA.

[3] Department of Chemistry, Washington State University, Pullman 99164, USA.

[4] Department of Microbiology, Immunology and Biochemistry, University of Tennessee, Memphis, TN 38163, USA.

Correspondence and requests for materials should be addressed to F.Z. (email: fang.zhu@wsu.edu) and S.R.P. (email: rpalli@uky.edu)

Additional Information

Reference

66. Mao, W., Schuler, M. A. & Berenbaum, M. R. CYP9Q-mediated detoxification of acaricides in the honey bee (*Apis mellifera*). *Proc Natl Acad Sci USA* **108**, 12657-12662 (2011).

Fig. S1 Summary of *L. decemlineata* transcriptomic sequences. The length distribution of Colorado potato beetle transcript sequences: (A) contigs; (B) singletons.

Fig. S2 Species distribution of the top BLAST hit in the nr database for each contig of *L. decemlineata*. A top BLAST hit is defined as the species which each sequence shares the greatest similarity to. The number of sequences for which this species was a top blast hit is shown on the y-axis. Singletons follow a similar distribution.

Fig. S3 Gene ontology (GO) terms for the transcriptomic sequences of *L. decemlineata*. (A) biological process; (B) cellular component; (C) molecular function.

Table S1. List for Cytochrome P450s identified in *Leptinotarsa decemlineata*.

Table S2. Amino acid sequences for Cytochrome P450s identified in *Leptinotarsa decemlineata*.

Table S3. Statistical analyses of five candidate reference genes based on their threshold cycle (C_T) value.

Table S4. Differential expression of 76 CPB P450s in imidacloprid resistant and susceptible strains.

Table S5. Induction of 76 CPB P450s by potato leaf GAs and imidacloprid in head, fat body and gut.

Table S6. Insect P450s selected for phylogenetic tree construction.

Table S7. Primers used for qRT-PCR.

Fig. S1

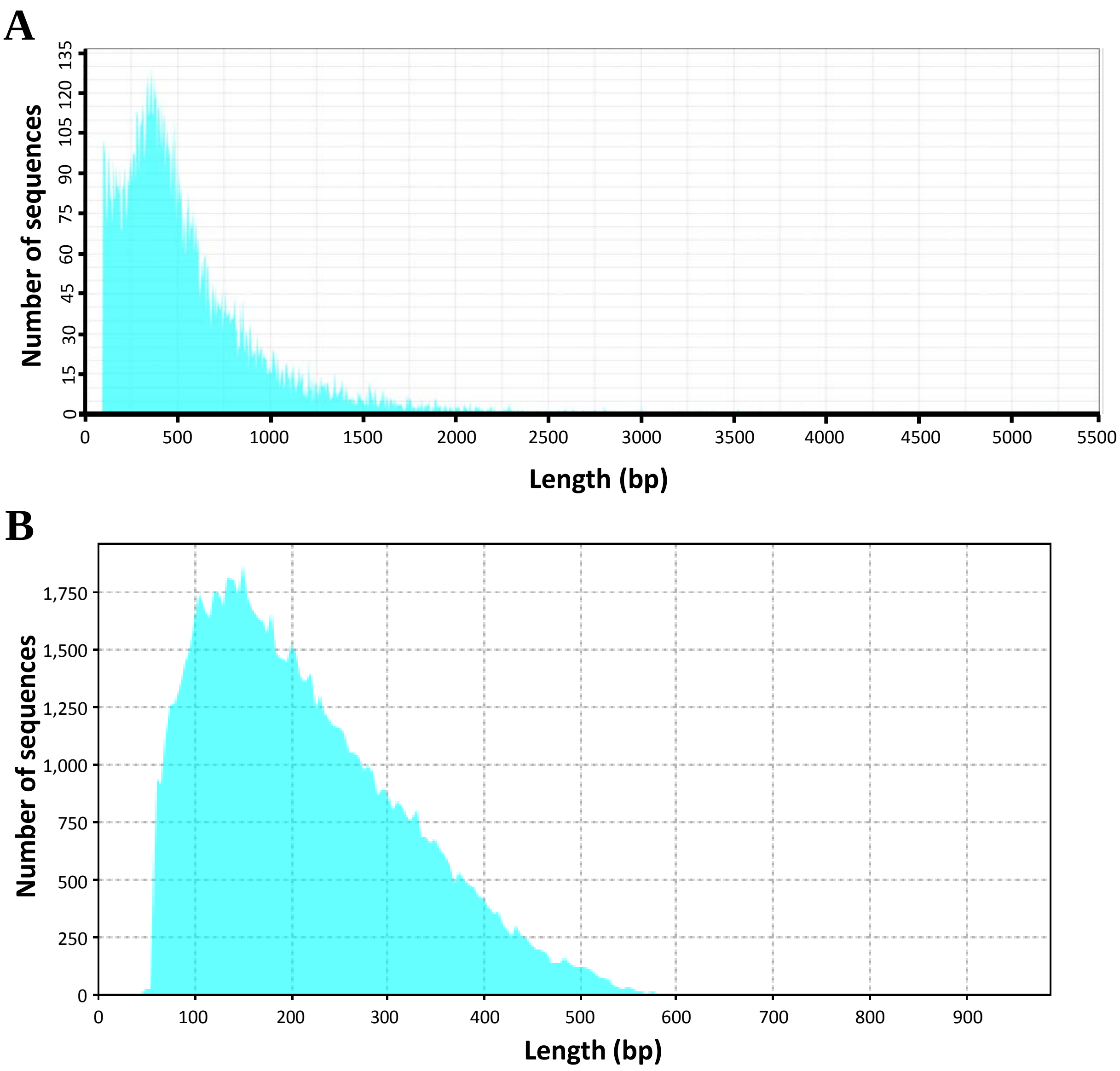
A
Number of sequences
0
15
30
45
60
75
90
105
120
135
0
500
1000
1500
2000
2500
3000
3500
4000
4500
5000
5500
Length (bp)
B
Number of sequences
0
250
500
750
1,000
1,250
1,500
1,750
0
100
200
300
400
500
600
700
800
900
Length (bp)

Fig. S2

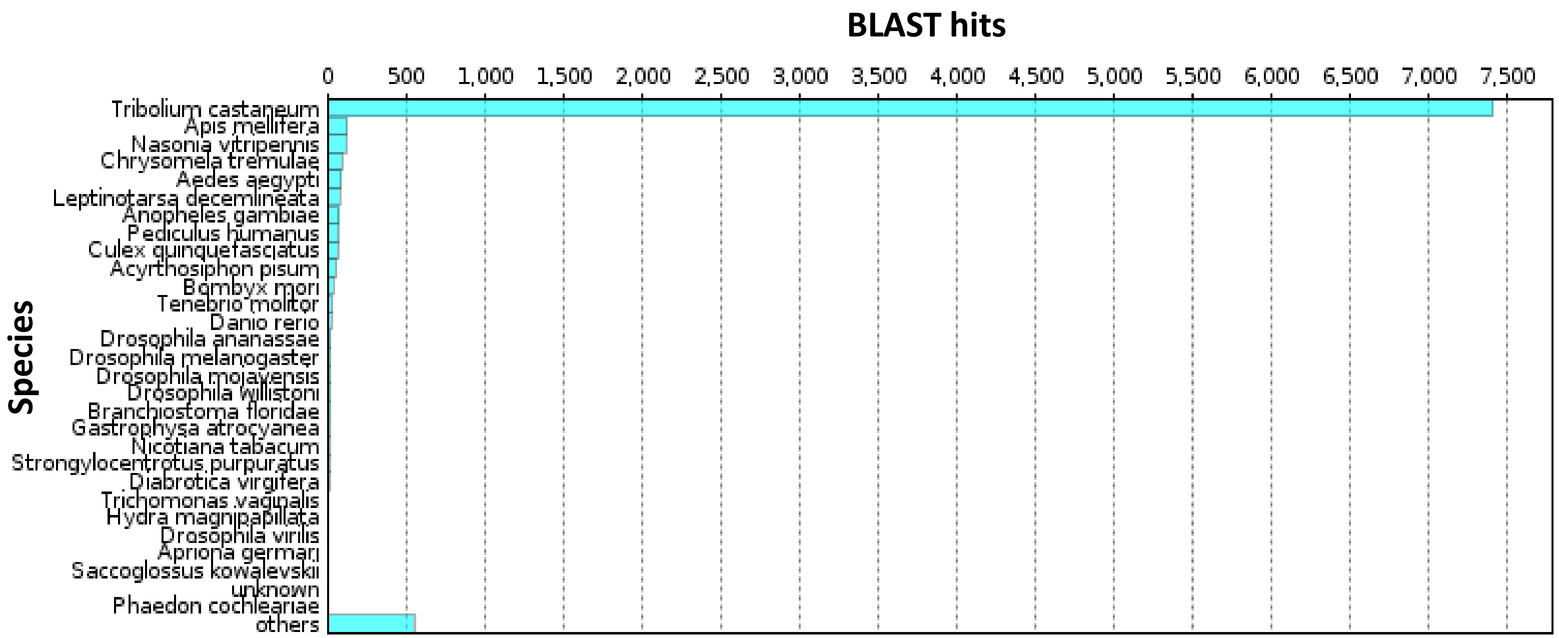
BLAST hits
0
500
1,000
1,500
2,000
2,500
3,000
3,500
4,000
4,500
5,000
5,500
6,000
6,500
7,000
7,500
Species
Tribolium castaneum
Apis mellifera
Nasonia vitripennis
Chrysomela tremulae
Aedes aegypti
Leptinotarsa decemlineata
Anopheles gambiae
Pediculus humanus
Culex quinquefasciatus
Acyrthosiphon pisum
Bombyx mori
Tenebrio molitor
Danio rerio
Drosophila ananassae
Drosophila melanogaster
Drosophila mojavensis
Drosophila willistoni
Branchiostoma floridae
Gastrophysa atrocyanea
Nicotiana tabacum
Strongylocentrotus purpuratus
Diabrotica virgifera
Trichomonas vaginalis
Hydra magnipapillata
Drosophila virilis
Apriona germari
Saccoglossus kowalevskii
unknown
Phaedon cochleariae
others

Fig. S3

A

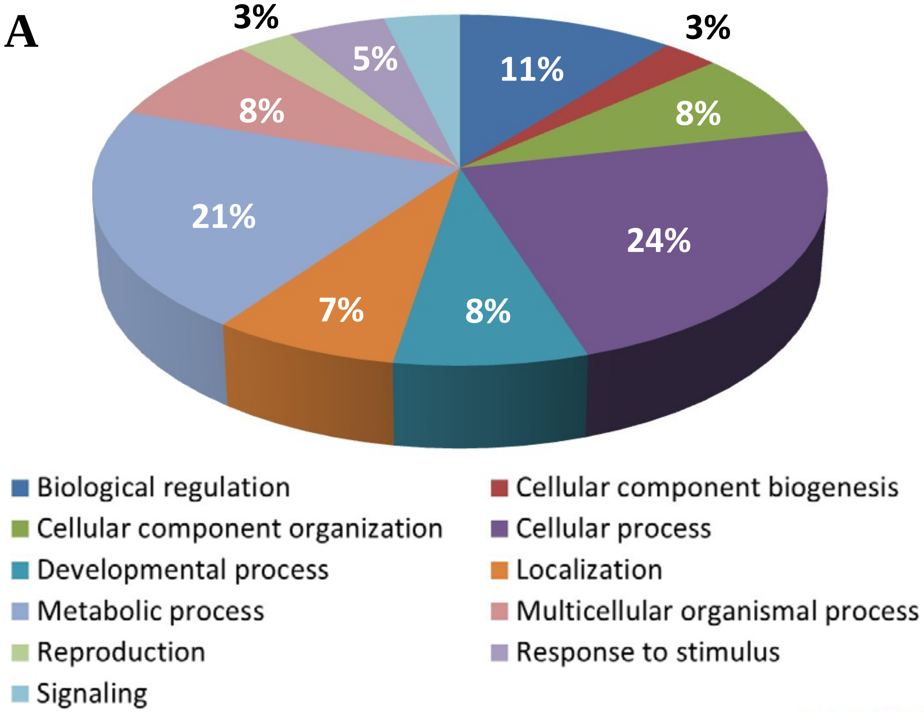

11%
3%
8%
24%
8%
7%
21%
8%
3%
5%
Biological regulation
Cellular component biogenesis
Cellular component organization
Cellular process
Developmental process
Localization
Metabolic process
Multicellular organismal process
Reproduction
Response to stimulus
Signaling

B

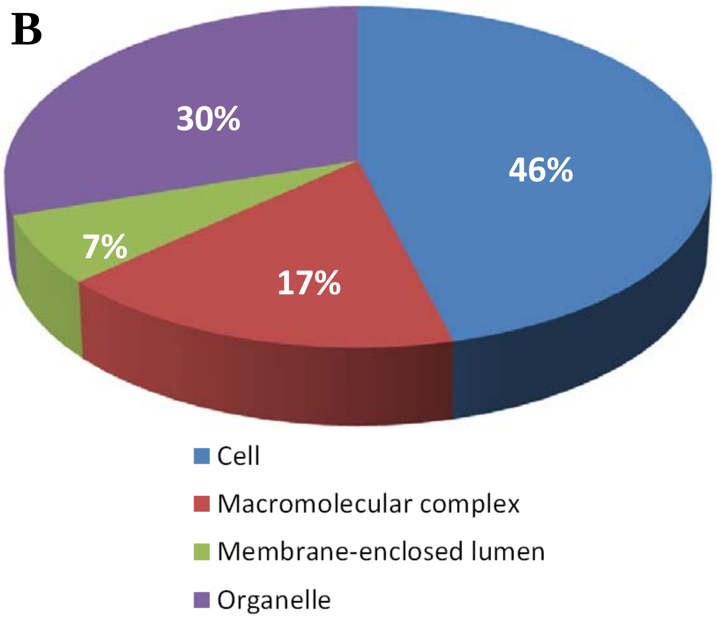

46%
17%
7%
30%
Cell
Macromolecular complex
Membrane-enclosed lumen
Organelle

C

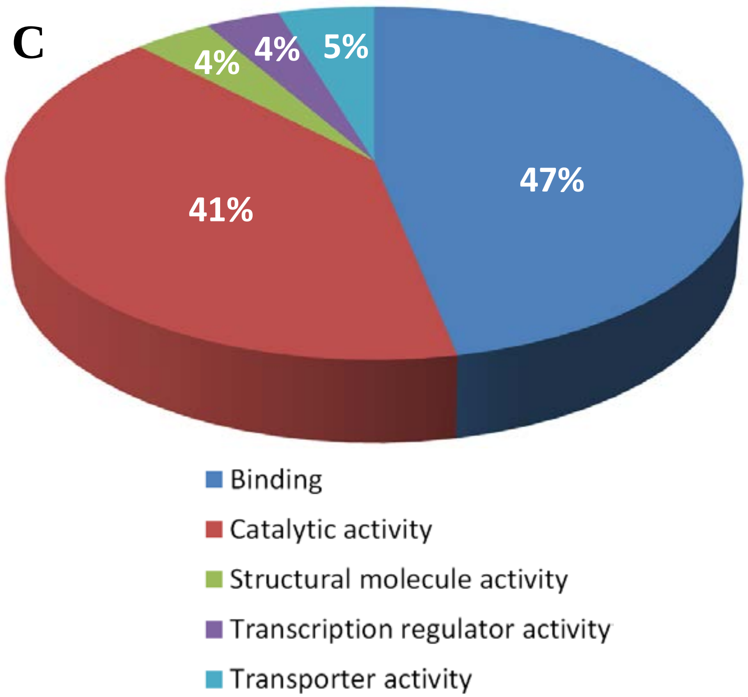

47%
41%
4%
4%
5%
Binding
Catalytic activity
Structural molecule activity
Transcription regulator activity
Transporter activity

Table S1. List for Cytochrome P450s identified in *Leptinotarsa decemlineata*.

No.	Clan	Primer No.	P450 Name*	GenBank accession no.[1]/contig no.
1	2	Ld96	CYP18A1	Contig00492
2	2	Ld110	CYP305A1	Contig03704
3	Mito	Ld122	CYP12H2	Contig06799
4	Mito	Ld9	CYP12H3	Contig04906
5	Mito	Ld128	CYP12J4	Contig09889
6	Mito	Ld8	CYP12J	EB758458[1]
7	Mito	Ld82	CYP49A1	Contig06413
8	Mito	Ld132	CYP301B1	Contig07011
9	Mito	Ld98	CYP314A1	Contig00869
10	3	Ld85	CYP6BH	Contig04303
11	3	Ld30	CYP6BJ1	DQ117463[1]
12	3	Ld113	CYP6BJ3	Contig12558
13	3	Ld15	CYP6BJ[a]	EB757598[1]
14	3	Ld84	CYP6BJ[b]	Contig10557
15	3	Ld106	CYP6BK	Contig07056
16	3	Ld154	CYP6BQ15	Contig08488
17	3	Ld143	CYP6BQ16	Contig01265
18	3	Ld27	CYP6BQ21	Contig00220
19	3	Ld148	CYP6BQ22	Contig01264
20	3	Ld161	CYP6BQ	Contig10876
21	3	Ld45	CYP6BU[a]	Contig09262
22	3	Ld157	CYP6BU[b]	Contig09308
23	3	Ld2	CYP6ED1v2	Contig01354
24	3	Ld104	CYP6ED	Contig10723
25	3	Ld5	CYP6EE1v2	Contig01835
26	3	Ld78	CYP6EE1	Contig03888
27	3	Ld12	CYP6EF1	EB757657[1]
28	3	Ld80	CYP6EG1	Contig02306
29	3	Ld6	CYP6EH1	Contig04994
30	3	Ld115	CYP6EZ1	Contig01667
31	3	Ld93	CYP6FA1	Contig00754
32	3	Ld101	CYP6	Contig07881
33	3	Ld126	CYP9A	Contig09946
34	3	Ld13	CYP9AV1	Contig00465
35	3	Ld29	CYP9V1v1	DQ117460[1]
36	3	Ld25	CYP9V1v2	EB754758[1]
37	3	Ld41	CYP9V2v2	DQ631662[1]
38	3	Ld83	CYP9Y[a]	Contig11158
39	3	Ld114	CYP9Y[b]	Contig12257
40	3	Ld117	CYP9Y[c]	Contig09375
41	3	Ld42	CYP9Z13	DQ631659[1]
42	3	Ld63	CYP9Z14v1	Contig01918
43	3	Ld38	CYP9Z25	Contig00260

44	3	Ld3	CYP9Z26	Contig00493
45	3	Ld68	CYP9Z28	Contig01161
46	3	Ld147	CYP9Z29	Contig01238
47	3	Ld39	CYP9Z30	Contig03170
48	3	Ld134	CYP9Z31	Contig01532
49	3	Ld152	CYP9Z[a]	Contig06948
50	3	Ld140	CYP9Z[b]	Contig03035
51	3	Ld40	CYP9Z[c]	Contig08090
52	3	Ld37	CYP9Z[d]	Contig04061
53	3	Ld121	CYP9Z[e]	Contig08039
54	3	Ld43	CYP9Z[f]	DQ631658[1]
55	3	Ld158	CYP9Z[g]	Contig10068
56	3	Ld156	CYP9Z[h]	Contig09087
57	3	Ld1	CYP9Z[i]	Contig06620
58	3	Ld155	CYP9Z[j]	Contig08801
59	3	Ld150	CYP9Z[k]	Contig06091
60	3	Ld97	CYP9Z[l]	Contig08836
61	3	Ld159	CYP9Z[m]	Contig10701
62	3	Ld160	CYP9Z[n]	Contig10810
63	3	Ld21	CYP9Z[o]	EB755804[1]
64	3	Ld109	CYP9	Contig11061
65	3	Ld20	CYP345G1	Contig00315
66	3	Ld74	CYP345G	EB759967[1]
67	3	Ld164	CYP345[a]	Contig12742
68	3	Ld129	CYP345[b]	Contig02242
69	3	Ld88	CYP347C	Contig02790
70	3	Ld105	CYP413A1	Contig09360
71	4	Ld131	CYP4AA1	Contig07817
72	4	Ld50	CYP4BN12v2	DQ631646[1]
73	4	Ld118	CYP4BN12v3	Contig01477
74	4	Ld48	CYP4BN13v4	DQ631649[1]
75	4	Ld49	CYP4BN14v1	Contig01451
76	4	Ld89	CYP4BN15	Contig00384
77	4	Ld153	CYP4BN	Contig07821
78	4	Ld32	CYP4G29	DQ117464[1]
79	4	Ld127	CYP4G34	Contig09239
80	4	Ld81	CYP4G[a]	Contig02645
81	4	Ld141	CYP4G[b]	Contig05926
82	4	Ld125	CYP4Q10	Contig04050
83	4	Ld46	CYP4Q11	Contig09919
84	4	Ld22	CYP4Q[a]	EB755786[1]
85	4	Ld166	CYP4Q[b]	Contig02266
86	4	Ld119	CYP4Q[c]	Contig03154
87	4	Ld138	CYP4[a]	Contig04089
88	4	Ld133	CYP4[b]	Contig06673
89	4	Ld123	CYP4[c]	Contig09902

90	4	Ld72	CYP4[d]	EB760335[1]
91	4	Ld107	CYP4[e]	Contig10575
92	4	Ld120	CYP349B	Contig08032
93	4	Ld142	CYP349C1	Contig05067
94	4	Ld35	CYP350D1	DQ631673[1]
95	4	Ld165	CYP350D2	Contig13007
96	4	Ld162	CYP350D4	Contig11512
97	4	Ld130	CYP411?	Contig05829
98	4	Ld7	CYP421A1	Contig02661

*Names here were provided by P450 nomenclature committee.

Table S2. Amino acid sequences for Cytochrome P450s identified in *Leptinotarsa decemlineata*.

P450 name	Amino acid sequence
CYP18A1	GGVVAKIEQNRVEMAEYMQEKIDDHKKTFDPGNPRDLLDMYVNDVSL AMKEGNVDRLFHGKDPDRQIQQIMGDLFSAGMETIKSSLQWSILFMLHY PDEMKAVQEELDEVVGRGRLPALDDISYLPITNATLNEILRIANIVPLGTT HAPTRVIKLGKYTLPKHAQVVPLLHYVHMNPDLWDEPEKFNPSRFISSE GKLLNPECFLPFGEGRRKCLGEVMARMEIFLFFSSILHSFDLSVPEDHPLP SLKGIAGVTLNPNAFKVVP
CYP305A1	WTYFWLGRQTTSNTLDFAFLMMLLYPEIKDKVHACLDEAFQKSEEITYS ERKRVPYVEAVLYETERFCTIAPIIGPRRVLRKTTLNGYDIPKDTTVLIHIH LVHNDVDYWQDPEIFRPDRFLDPKGNLMYHDRFLPFGLGKRRCLGEILA KNCIFTFFSQIMKSYQIELPPNAKKPTGVPQPGITLSPEKYRAIFIKR
CYP12H2	LFLPWSTPITTTIRALHPAIEFRYISCTCLPFFCFFGGTLTAMLIRCASRPLA LGARAVSASSSKPCAGAAAVSKREDYYKTLGEHVDESKPPGWDSARPFE EIPGPKPLPIVGNLLRFMPFCRGAVQDSNLGDVPKIQGTIWRRGN
CYP12H3	MGTFTAMFTKYASRSFLLRTRKVSSKPGAGPAAVSKRDDYYKTLAEDT NESKPPGWDSALPYEKIPGPKPLPIVENLRRLLPVVGELPKSPILKMLNQS REQYGDVVVWKNILGKPDSVYIYNIKDIENLLRNSGPYPVRTVLDIFVYY RTVLRKEIFQDVGGVLTVQGEDWFKIRSVVNPILMQPKA
CYP12J4	RAGHFKDPEKYMPERWLRDTNDEYSSKNVHSFASLPFGFGPRMCVGMR FASLELELVLMKIIRNFELSWEHPDMEFASHLLYGINNPLKLTVKELTR
CYP12J	ESCIKESTRIAPVTIGSFRTTVKDLVLGGYQIPKGTNITVVSICTSNSSEHFK DPEKFVPERWLREIDDDYSKNVNPFASLPFGFGPRKCVGMRFANFELEL VLMKIIRNFELSWEHSDMEFASHLLYGISNPLKITVKEVTR
CYP49A1	NLAKMSSGATRKLLAPKVIRAFKRSYSKDRPYSTALGVMPRVYEDDIYE YKVEKVDVITKDYSEIPGPKELPLIGNAWRFAPLIGQYKIHDLDKVMWSL RKEYGKIVKVGGLVGHPDLLFVFDGDDIK
CYP301B1	QHYVISNLEQYFPRSKEFLPERWLKSCPFSKEQHPFASLPFGFGKRMCLG RRFVDLEMQTALAKIIRTYKIEYHHEKLDYFVHPMYTPNGPLKLKFIKHG S
CYP314A1	MARVTDFSDKKLTSPKTVAGFLPQVQEIVEDWCNLISQQRTDNTITNLED IVGPLGLEISCALVLGRRMGFLLPGAESETAQKLAETIHQLFIATRDTYYG LPFWKIFETPAYKKLAESEDTIYQLVTNLISTADQAANRSPVFQSVLDANI NQKEKTAAIVDFIAADIHTLKNSLVFLLYLVAKNPGTQEKILEDSSKSYLR ACIMETFRIYPTANCLAR
CYP6BH	NSITMNYLLELLIFLVTLSISVTIFVHYRYGYWKRRNVPYLKPIFPMGNNT SFFPKEYPLELYPNIFTMNSRRWDARSEVYSLGSIRNWLSWIRTSPKIF
CYP6BJ1	MVFSTDNLLLDLFGVLVALSAVLFVYIKRSFQYWDRKGVPYLPPNIPWG NLQPPHSRDIPEGDDVANIYYKAKAKGWKYIGIYVMTGSVFLPVDLELIK HITTKDFQHFVDRGTYVNEKDEPITAHLFSIGGKKWRNLRTKFTPTFTSG

	KMRQMFETIANCGHILEKYIEHEVDHHEPLDIKNVLACYTTDIIGSCAFGL<br>DCNSFKEPNSPFTQFGQRVFRTDGIRNLKITFMGAFPNLSKMLRMRLTEK<br>EVEDFYTKVVEDTVRYREKEGVTRPDFLQMLIDIKNKTNEHTGDGTSLT<br>MDEIVAQSFVFFIAGFETSSTTMTFALYQLATHPEIQEKVRSEINSVLEKH<br>NNQITYDALNELKYMGKVIDETLRMYPALPVVTRRCVEDYRIPDSDVIIE<br>KGIEVFIPIKAIHYDPEYYENPEVFDPERFNEENIQGRHPYAHIPFGEGPRIC<br>IGLRFGVMQSKVGLVSILKNFRVTLSSKTKLPLKIDVNSFIPTTEGGMWL<br>NLERIGK
CYP6BJ3	ERPKDFQHFVDRGTYVNEKDEPISAHLFAIGGKKWRNLRTKFTPTFTSGK<br>MRHMFETIANCGLILEKYIEHEVDHHEPLDIKTVLACYTTDIIGSCAFGLD<br>CNTFKEPNTPFTQLGQRVFQTDRIRSLKIAFMMAFPDVSKMLRMRLWPL
CYP6BJ[a]	EPFRKIMVSFTDNLLLDLFGLLVALSAVLFVYIKRSFQYWDRKGVPYLPP<br>RIPWGNLQPPHRRIIPFGDDVANIYNKAKAKGWKYIGIYVMTGSVFLPVD<br>LELIKHIMTKDFQHFVDRGTYVNEKDEPISAHLFAIGGKKWRNL
CYP6BJ[b]	GVMQSKVGLVSILKNFRVKLSSKTKLPLKIAVNSFIPTTEGGMWLNLERI<br>AK
CYP6BK	KNEISSNGPKLTMNNVAALSFGLFIGGFETSTSSFTFCMFELASHQNIQDK<br>VREEVKTVLARHNNEFSYESLGEMKYLRQVLDENWRLHPPAHTINRRCT<br>KDYKVPGEDLIIEKGTNVLVTITGIHRDPEYYPNPLVFDPDRFSEE
CYP6BQ15	EENKSKRHPFAFLPFGEGPRICIGARFGLLQVKVGLTAIIRNFKVTLNENK<br>TP
CYP6BQ16	QIMTFLLTVTYILGAVIVGIYLLLKWIYSYWYRKGVEYIEPDFFYGNVKE<br>MIQRKVSLGGLFKKFYDNLKSRGLKYGGCYTFFSPVFIPVDLDVIKSILLK<br>DFDHFVNRGMYYNEKVDPLSTHLFTLEDERWKSLRSKLTPTFSSGKLKM<br>MFPTLVSCSFGLEDVLNEYSAIQDAVDIKEVSSRFSTDAIGSVAFGIDCNS<br>LKNPNSEFRQWGRQMFLGGIRGIIKAIMLMTLPNSFLHVIRFKMTSKSTE<br>DFLMNVVRDTVDYREKTMCIEKTSCIYYYS
CYP6BQ21	ESQVWWNIRFFSPVFIPIDLNLVKSIMLKDFHHFVNRGGYVNEKADPLSG<br>HLFSLEDEKWRNLRAKLTPTFTSGKLKMMFQTLVACTSGLEDILSEHSIL<br>QDAVDIKEVSARFTTDAIGSVAFGIDCNSLKNPDSEFRQWGRRIFKGSIRG<br>QIKALMLMTLPNNFLHAIGFKLTNKTTEDFLMNMVRKTVDYREKNNIYR<br>KDFMHLLLQLKNRGKVTDDANITKEEEKNKGSDTLSFNEVAAQCLVFFI<br>AGFETSATTMTFALLELSLNQDIQDRLRQEINMVLKKHDMQFTYDSVME<br>MKYLEKVIDETLRKHSAATVLRRVCKKAYTIPGTNVVLDEGTAVNIPVL<br>GIHRDPEYYPEPDKFDPERFNEENRARRHPFAYMPFGEGPRICIGARFGLL<br>QTKIGIAAIIKNFKVTLNEKTKTPIRYSPSARITGVDGEVWLNVTKIQ
CYP6BQ22	YSHNNDVSANIDFYFGRIDSWRLFIHKTDIFLLVPERSGVHRTRLFYGNIK<br>EMVHGKISTGGLFKKFYDDMKSRGLKYGGCYTFFSPVLILVDLDLIKNIL<br>LKDFDNFINRGMYYNEKMDPLSAHLFSLEDERWKNLRAKLTPTFTSGKL<br>KMMFPTLVACSFGLEDVLSEYSVIQDAVDIKEVCSRYATDAIGSIAFGIDC<br>NSLKNPDSEFRQWGKQMLKGA

CYP6BQ	DMQFTYDSIMEMKYLEKVVDETLRKHSAGTILTRICNKPYTIPGTKVVIE<br>KGTAVYIPVSGIHRDPEYYPDPDKFDPERFNSENK
CYP6BU[a]	ARHPFAYLPFGEGPRNCIAARYGLLQTKVGISAVIKNFKVTLNKKTKTPI<br>RYVTSGRIPEIEGGVWLDVTKIQ
CYP6BU[b]	ARHPFAYMPFGEGPRMCIGARYGLLQSKVGISAIIKNFKVTLNDKTRTPIR<br>YAKTGIIPRIEGGVWLNVTKIQ
CYP6ED1v2	SYNFIGSMEQYFFNLLFKLISSVGIVFFILYCFFLSYYKYWEKRNFPYLVP<br>RFPLGNSQSLFCKFPGPNLEFTDHYREIKKKGYKFAGVFATIRPILVIADT<br>EIVKDILVKDFDHFADRGFYKTASDPLSVTLFSMDAVEWRKTRIRLTPTF<br>TSGKLKMMFPLIICVSASMIEEIDDLAKENSDIDIKKIATNFTINTLAICTLG<br>IEPGKSESSYILSEMAQKHFDVHTPIDLLRYSLMNRFPDFSSILGLKLLKKE<br>VSEFFITVVRDAMKYREEMKLFRPDLLELLMNIKNETNNSPNPLTDNQLI<br>AEIFDFLSCVTRHIFSHNFFHFYSN
CYP6ED	PRNCVGNRFGIMVTQVALIEVLRMFRISVSSSTILPFEMDKQTLILVLKNPI<br>LLKAEKINNECDNLMI
CYP6EE1v2	YQRRYMGVHLRLLSLHSDCDLFFINFIVVPGIMELLIFDYLTKTLVYSSIL<br>ATFIYLYFSWCYGHWKRRNFPFLKPNFPKGNNPTYLARFPGPHLECRDH<br>YLEIRKRGLKFAGIFCVIHPILVVVDPVIIKDILVKDFEHFVDRGFYSSEND<br>PLSVSLLSMGGDKWKNSRGKLTNFFTIGKVKMLIPLMVGSAKPMVRSIR<br>ECASNNTDINITLLTKRYAAETVGTCFLGIDCNNFGDVEDTFANMVGKFF<br>RDQNFTHLLRVSCTNRFPALSRKVGLKLILSEVSEFFKSAVRDTLKYREE<br>HNYFRPDVLQLL
CYP6EE1	KLSGILRPGLPSLERECVKDYKINGTNLTIEKGTTVWISIMGIHRDPEYHT<br>DPEKFDPDRFIEDSAKSYSQFYFPFGLGPRMCIGVRFGLTVAQIGLIRILRE<br>HRMSLSDETCTPLKLNKQSFLLETLEPLLMRAEKI
CYP6EF1	GPIKKSIMGILSVEILGTFSMVMLICFSTIYLYFNISYQYWKRRGVPYLKP<br>KFPTGNSSSLFRKTRVFPLATMDFYKEIKKRGWKFGGVYTVLRPVLVIV<br>DPALMKNILLTDXQYFIDRGFYYNEKDDPISAHLFALDEKSWKNMRIKL<br>TPTFTSGKMKMMFPACS
CYP6EG1	VEPRLIEKTMFILPLLSHLVIVIVFLILVLIAFYKWSFLYWKRRNIPFLEPSIP<br>FGSLMNMLTRKISRIEELTMMYNECKKRGYKHCGIWQLADPTYFVVDL<br>ELLKNICIKDFEHFADRGFYFNEKDDPISAHLLSLDGDRWKTLRKQSSTIF<br>SGAKLKGMFPNVLACAESLENYVASLVENGSQPVNVKDIASSFAVDSVG<br>SSTLGIKCRGFTDPDKKFIHYSKQALETDRKSQLRMLFGIFFPNAARKMG<br>MRQQNKDIGDFFTNVICDTVQKRKAQNVERND
CYP6EH1	AFVQCFHSYCGVESFLIQVSQSVRELNMFDLSPSTLFLLGIFICLSGYLLW<br>FFYEHHQYWKRRNVPYLTPTIPFGNTLDLFRGKISFGEVFSRAYLELKKQ<br>GHKHGGIYYLQKPVYIPVDANIIKKIIISDSDNFPNHGMYVNPADDPLSGH<br>LFNIEDGRWRTLRSKM
CYP6EZ1	HGQWNKYKNSMVVAVPRHILEKLSFRIFSKETEKYVLKMFKDILRYRKF<br>DSPLREDLTASVVKLTEWREEEKDFSGKKVMVPLEESEFAAHMFLFFCA

	GFETSSSTQTFALFELARNPECQNKLRKEINTVMAKHGNEVTYDAIMEM<br>EYLDNIVDETLRMYPVFPILPRICKEDYPIPGTDLILEKNTFVMVTNLGIQR<br>DPEYYPNPEQFNPERFNREIRESFHISQIYLLVKDPEFAWEKDSVCGKPN<br>WA
CYP6FA1	SSSTVFFFFLAGFETSSTTICFASYELALHKEIQDRARHEIKEVLKKYNNEI<br>TYDALMEMHYLETVIYGTLRKYPPLQFLLRKCTDNYKVPDSNIVIEKGM<br>VTIIPVLGLHFDPEYYPDPEKFDPERFNAENKKTIPQFAWLPFGEGPRICL<br>GLRFGMVQVKIGLISLLKNYEFTLHEKTISPIVYTKTSVLASAKGGIWLNL<br>RKIH
CYP6	TGSAVWYTTRKIFFRTMSITGYFFHDLVLVWISLMIITITYYKHAFKYWS<br>QRGVDGPEPTVIFGNGWDVANGSKTMGELWKKIYLELKSKGLRHGGGY<br>FMAKPMYTVVSLDLVKAIMQTDYQYFN
CYP9A	LLVPLACNATPSKNDKNDFFTMGSRMTKPTGFNVIKGILAAFFPHVFEFF<br>RIPIFPSFVTKF FQDLIKETISVREKIISSAQI
CYP9AV1	LFRQDVLRINVDSFAEPNNEFWQMCHKAVDTISKPKVLSFNVYLLAPKL<br>VKFLKMKIVDWDADSFFRQIISDTMKIREKERENGILGADLIHLLLEGRK<br>RAKMNKDKETNVKSTYKNYNADWSNMDIIAQAFFFHFASCAPAPTLLSF<br>MGYELAVNPDVQEKVREEIRETDEICGGKLTYEVLNRMKYIDMVVQES<br>MRKWPSVIEVDRICSKDYVIQPVLPEEKPVLVKRGSRVIVPIYSLHHDPQF<br>FPEPERFIPERFSDENKHKIKPNTFTPFGFGSRTCMGSKYSILQAKL
CYP9V1v1	MLTTIVLALLAVTAFYYLFIRPLSYWKKRNIKQGNPVPILGDYWWFFFK<br>MESFADLVRRLYNQFPNERYCGIYQYVTPTLMIRDPELIKQITVKDFDHF<br>VDHRAVMPETSDPLWAKNLFSLTGQRWREMRPVLSPAFTGNKMRLMF<br>GLISECADDYVSFYLEKNQDVIDVELRDTITRFTNDAIATTAYGIKVDSLR<br>NPANDFYVKSRKAFTLSSFLALKFFSTLLLPTFIVNLLNLKIFEKEISDFFIT<br>IVDETIKIREEKGIVRPDLIHLLMEARKGHYRYEEESGTTDTGFAAVKESD<br>IGKHIRQKIEITNLDIAAQALVFFLGGFDSSASLMCFMGYELAVNQDIQRK<br>LRIEIEDTLEKCNGVITYDALLKMKYMDMIISETLRKWSNGVIADRVCTK<br>PYTIEPVTAEEKPIHLAEGTFIIIPSFGIQHDPKYFPDPDRFDPERFNEENKD<br>KINSYTYLPFGIGPRNCIGSRFALLETKLLFFKLLSKFEIVPTTKSGIPLKIST<br>TTLNLNSEGGFLFAFKRLNGNQ
CYP9V1v2	NMLTTIVLALLAVTAFYYLFIRPLSYWKKRNIKQGNPVPILGDYWRFFFK<br>MESFADLVRRLYNQFPNERYCGIYQYVTPTLMIRDPELIKQITVKDFDHF<br>VDHRVVMPETSDPLWAKNLFSLTGQRWREMRPVLSPAFTGNXMRLMF<br>GLI
CYP9V2v2	PFTAGPRNCIGSRFALLETKLLFFKLLSKFEIVPTTKSGIPLEISTTTLNLNS<br>EGGFLFAFKRINGNQ
CYP9Y[a]	FSKFIRPVLYWKKKNIFYVTPWKGFLRVLFGPKSFFELIVEAYRRFPEKRY<br>YGSYQFLNPSLFVTDLDLIKQITVKDFEYF
CYP9Y[b]	NGVNRRSDHSSGSGFLFAGFDTSSTLLSFLSYHLAVDQKIQTTLQKEIDN<br>VTKLGDGRVSYEELLKMKYLDQVISETLRRYPPGFMLTRICVAAR

CYP9Y[c]	LSKFDIIVIKKTPFPLLLSKTFNLAGREGIWLGLKKREKF
CYP9Z13	PFGEGPRNCIGSRFGLLEIKTVFFHILSHFELIPTEKTPIPLKLTKKTFNLGA<br>EGGFWLGLKRLKK
CYP9Z14v1	YVIEPTNPEEKPLYIEKNTLLMIPIVGIHRDPKYYPEPMRFNPERFSDENKG<br>KIDPFTYLPFGLGPRNCIASRFALLEAKVVFFHLLSHFELVPVEKTPIPLKIS<br>KQSFNMVAEGGFWLGLKRLKK
CYP9Z25	RCRTSVEQKFICINRKKWRDMRPILSPTFTSSKMRTMFLLISDCSEKFVKH<br>FVENNENCIEIEMKNIFTRFTNDVIATTAFGVEVDSMKQPNNEFYLMGKE<br>VNNSTGFWRLLKIFGYFIIPSVYHFFRISVSPFTKKISMFFRELVDDTIKFRE<br>ENNIVRPDMIHLLMESRKGIEHKEENVDIGTGFATVEESFMGKGISGKEIT<br>NTDITAQALIFFFAGFEQVSSLMCFMALELAIHDDVQTRLREEIKKTISSC<br>GGNLTYEALMKMKYMDMVVSETLRKWPTTVLTDRICTKAYIIEPQNPG<br>EQPLHLERNANITIPMFAIHRDPKYYPDPDRFDPERFNDENKLKINPYTYA<br>PFGFGPRNCIGSRFALLETKLIFFHLLSHFELVPVKKTCTPLRASKKSFNLV<br>ADGGFWVGMRKLTKSMKSTLSTHT
CYP9Z26	MIQLVLVAAVLAALLYYFCIKPMNYWRERGVKQNDHPVWLFGENWRT<br>TIRKESFFELVVRCYNQFQGTRYHGIYQLIQPTLLIKDPELLRKITVKDFD<br>HFTDHRSFFSEETDPLWGKNLFTLRGRKWREMRPILSPSFTSSKMRTMFV<br>LISKCSKDFVNHFLKKDQDCVEMEMKDTFTRFTNDVIATSAFGVKVDSL<br>EEPNNEFYSMGKRATDFSGFAMMLKFIGFFVAPDLLKYLRISFMDSAVN<br>RFFCDLVDNTIQVREKNNIIRPDMIHLLMEAKKGVHHKDENSGVDTGFA<br>TVEEADLGEGHVTEITNLDITAQALIFFFAGFDSVSSLMCFMSHELAVNP<br>NIQTKLRNEIEETLAGCGGEITYEALLKMKYMDMVISESLRKWPSAVAT<br>DRVCTKPYTIEAMNSDEKPLLIEKGTTLMIPIVGIH
CYP9Z28	MFLIIIGTAILAALFYYVCIKPMSYWRERGVKQTDPIWVFGDNWMTATK<br>KESFLDFIIRCYNYYPGARYYGVYQFLQPTLLVKDPELLKKITVKDFEHF<br>TDHRVLISEDSDPHFGKNLFALTGKKWRDMRPILSPSFTSSKMRAMFVL<br>MSKCAEDFTNHFLKKDQECIEMEMKDTFTRFTNDVIATTAFGIKVDSLEE<br>PNNKFYLMGKETTDFSGFWKSLKMTGFFLVPKLLQYLRISFLSRSATQFF<br>SELVDETIKTREEKI
CYP9Z29	MIELFLGAAALATIFYYFYIKPMNYWGERGVKQEGHPLWLFVDSRKSKY<br>GKESVFDSVVRSYNQFQDTRYHGIYDSTRPILIIKDPELLKQITVKDFDHF<br>TDHRNHIELDVDPLWSRNLFFQKGEKWRKMRSILSPSFTSSKMRIMFVLI<br>SKCTENFVNHFLKKDEKCIEVEMKDTFTRFTNDVIATSAFGVEVDSLEEP<br>NNEFYSKGKKSTTFEGVSKLFMFIRYPLVPKLIEYLQIPFMDPVVYRFFCD<br>LVDNTIEVREKII
CYP9Z30	EDITAQALVFFFAGFESLSSVMCFMAYELAVNQDIQEKLRMEISESFEKC<br>GGELTYAVLVNMKYLDMVVSEVLRKWPTQTAAERVCSEAYTIIPTTPEE<br>QPLHLEKGSIIVFPQFGIHRDPKYFPDPERFDPERFNDENKSNIVPFSYQPF<br>GQGPRNCIGSRFALLEMKVLFFYLLLNFEIVPIERTRIPLVLSKSSILVSVE<br>GGFWMGLKHIEK

CYP9Z31	MMLIIGILVAVVILLYLLLVKPLSYWNEKGVKQESILRALSFNWFSIFGRQ SVALYVKNMYDQFPNERYSGIYQMNAPVLVIRDPELMKLILVKNFDHFT DRRTYVPEDMDPLWAKNLFALKGSKWRNMRSTLSPAFTSSKMKRMFILI KDCAESLVEHFSKQNQEIISLDMKDTCTRFCNDVIATTAFGVKVDSLADP NNEFFLMGMKATNLNTLKKTFQFLGYLLAPKVYRLFNLKFFDHDVSSFF RKLIDETVEAREKRGIVRPDMIHLLMEARSGIVKEEEISVEETGYASVQES VEGDTMTNIT
CYP9Z[a]	DEKPLHIEKGTLLMIPMVGIHYDPKYYPDPERFDPERFSDENKRKIDPFTY MPFGLGPRLCIGSRFALLETKLLFFHLLLHFELVPVEKTQIPLKLSKLTFNL TAENGFWLGLKRLKK
CYP9Z[b]	SFIENEIYGYGCIRIVEKMAKPIATDRVCTKPYIIEAANPDEKPLHIEKNTL VVISIIGIHLDPKYYPEPLRFDPERFSEENKGNINPSAYMPFGAGPRNCIGS RFGLLEIKTVFFHILSHFELIPTEKTPIPLKLTKKTFNLGAEGGFWLGLKRL KK
CYP9Z[c]	DPKYYPDPERFDPERFNDENKGKIDPFTYMPFGLGPRNCIGSRFALLETK LLFFHLLSHFELVPVEKTQVPLKLSRKTFNLTAEKGFWLGLKRLEK
CYP9Z[d]	DPKYYPDPERFDPERFSDENKGKIDPFTYMPFGLGPRNCIGSRFALLETKL LVFHLLSHFELIPVEKTQIPLKLSRKTFNMMAEKGFWLGLKRLKK
CYP9Z[e]	FYILLNFAIVPVEKTKIPLVLAKSMFHLGAEGGFWLGLKRLKK
CYP9Z[f]	VHIEKETLIMIPIFGIHRDPKYYPDPLRFDPERFSDENKGNINTFAYMPFGL GPRNCIGSRFGLLETKLIFFHLLFHFELVPVEKTSIPMKLAKGTFNLMAEG GFWLGLKRIKK
CYP9Z[g]	PRYHSSGCAIFFAGFDTVSLLMCFMSHELAVHPDIQTKLRDEIEETLAECG GEVTYEAILKMKYMDMVISESLRKWPSTISTDRICTKPYTIEAKNA
CYP9Z[h]	YYHILSHFEIVPVEKTKVPLEVAKIPRLNAEGGLWVGLKRLDC
CYP9Z[i]	DPERFSDENKGKIKPLTYQPFGHGPRNCIGSRFALLEAKVLFYYILSKFEI VPTEKTTIPLVLANNIVVLAVEKGFDMGLKRLEK
CYP9Z[j]	RINLKTMLLLLIGLLVVAILVYFSLVKPLSYWKKKGVEQENFLKAFWFN WFGICGRQSLVSYVQNMYYQFPNRRYSGVCQLNVPVLVL
CYP9Z[k]	DICKMIQWFLGAAALAAIIYYFCIKPLSYWRERGVKQNDRPTWLLGDDW KFIIRKESFFEFTVRCYNQFQGTRYYGIYESGQPTLLIKDPEILRKSLLKILI TSPITETLFQRRLIHYLAK
CYP9Z[l]	MLQLLFGVAILAALFYYFCVKPLNYWRERGVKQTSKVWLFGDNWSTTI GKESFLDMFVRCYNQFPDSRYHGIYQF
CYP9Z[m]	QKCIEVEMKDTFTRFTNDVIATSAFGVEVDSLKEPDNEFYLMGKEATDL GGFGIMFKFIAYSLVPELMKFLRITLLKQAVIKFFCDLVD
CYP9Z[n]	IVVIEMKDTFTHFCNDVIATTAFGVEVDSLANPDTEFYRMGKKATDLSRP GKQLRFIINIVAPWISKVLGVKFLKMTLERFRKPYNW

CYP9Z[o]	FTEKMLVPVILGAVLVLTYIYFNLYRPLCYWQNLGVKQDNPLTLLKKLL<br>LTFYNVIFKREDPLEAVINRYDKFPGSRYYGIYEMTSPGLMVKDADLVT<br>QMLVTDYVHFNDHRVLIHDDLDPLLPSVFLPSKEKNGXR
CYP9	PRRYLPFGIGPRSCIGSSFRISYGSNSSYRNFEKIQNRCEPINMFAYGYG
CYP345G1	VGDGSLKIVHRAVFKKVVWFSFQFENYDKMFPSSSNFSVDISICLSTIIILL<br>YLYWTRNFNYWKNRGIPYKKPIPFFGNIKDVLTFRKSIGMNLKDIYDEM<br>DGPYLGIFIMDKPALLLKNLDVIKNILVTDFNNFCDRTLTQRKSDLGSSLL<br>PLMKNPDWKAYRKLLTPAYSSGRIKTMYNIVVDNGVELVRHIAKIMEKT<br>DVMDARHEATLYGAEVITSTAFGLKGDCFEDENSGFKKAAERIFDFNDR<br>SRAIATSCYMMFHSLVHIFRLKFVEPRSEKFLKDEFFKTVKYRQENNITRS<br>DFVDILMNLKKNNKVNEVQMDDEKMVAQAITFFIAGFETTSATISFSLYE<br>LAKNPEIQEK
CYP345G	KMFSIPTLTIYITICLSAIIILLYLYWTRNFDYWKNRGIPFEKPLPFFGNLKD<br>VLTFKKNIGMSPKDIYDKMEGPYLGIFLVDKPAL
CYP345[a]	GFETTSSSISFTLHELCLNREVQNKVRAEILETIKNHGGITYESIQDMKY
CYP345[b]	MLLIPSWILQLIIFIVFLICFLWMYSVRNFDYWKKRGVYSPKPTPFLGNIG<br>ELVFLKKCLSEWLSSLYFSTDERFFGVFMFDEPSLVLKDPKLIQLVMMKD<br>ADYFPDRSNAEPQDEIMSNFMFFKKVLAGKVIEQN---<br>NKIDSCFTSGKLKAMFSLMHNVAEEMVKYLEDNVGKIEAKDISARYSTD<br>VIAKCAFGIDAYCFDDQESDFRKYGRLMLEFSLRNAFSQMSYFFIQTWV<br>KIFHINLFSEEVRNYFSQAFTQTMKSRELSKTRVNDFVDLL
CYP347C	ARSVLRGTLIRQQIHSQKIYPLLWNQGLRSFLPVGALQNDPKYFKDPEKFI<br>PERFSSKENYNRYTYFPFGEGQRECLGKRFGTTQIKIGVAHLVKNFQLTV<br>DRKTQLPLKFDPLYFLISAVGGLWINVRKIE
CYP413A1	ISRWKHRRDLVNMSYCSPYDKTSFLYYVDENKLFSGITFFTFAVIGVFCYI<br>LRCYSFFEKHKIPRAVTPFAPYGNI
CYP4AA1	TSRIINIIMKYLLASSAVVILFLIYIRTYLRSVYLALKLPGPKALPIIGNVLFL<br>NDDREMEHLGNKAHELYGSFMRFWVSFLPCIVVHEPRHLNIILGTNRYS<br>RK
CYP4BN12v2	AGTRNCIGQKFAMLEMKSIISKVLRHFEILPATPEHKLKLAPEIILVSKNG<br>VCISLRKRFEL
CYP4BN12v3	VSINAQENSHSEYVRSVKEICAIVISRIFSPLDPRLYPLTWTSIREKIAVKKL<br>HAHTNFIIERRMKKVAVNGIDDGIDDVGERRNWRFWTCC--<br>RAPSRGNPCQWQTLGRKLIRLCLRGMILHHPPFLLRYIVWQHTQKCRKK<br>LQKNKKRLFGNLKEVRPKAQDLNNMKYLELVIKEVLRLYPSVPFLGRKI<br>PEDFEWGGNNLSERSEYPVAYICKSSESQLFPGALEIYSRKIRKL
CYP4BN13v4	QKFAMLEMKCTLSKILRNFELRPATPKHTLKLTPEAVLKSSNGVRIQVRP<br>RKL
CYP4BN14v1	KRMAFLDLLLQTPDETLTNEDIRQEVDTFMFAGHDTTASAVSFIIYCLAE<br>HPEHQEKSWKNWKPFLETTNLGR---

	QSRICKSMKYTEMVINETLRLYPSVPFTGRILNKDVKYEDGKILPEGLSLI<br>LYIYGANRNGQYYEDPNTFNPLRFESEKPAPYSYIPFSAGPRNCIGQKFA<br>MLEMKATVAKLLLKFEIVSCGHVPILAAETVLFSKNGVKVTLKTRM
CYP4BN15	MSFVLGTIFAVIVAVLGYRWYNLLSIKQKLNWVTHIPGWPLVGNALEFG<br>GTEVLLSDMSRLFKKHGKILFIDFVGKANIFTVDYDFIEFVLGTHYILDKS<br>RDYRFIRNWLGTGLLTSDGPKWKGRRRIATPAFHFSILEQFVEIFEKNGNI<br>MINKLKKEVDKDSIDIYPFITLCALDIICETAMGVPVDAQLKENSQYVKN<br>VKLMCKIGVLRSTSILKHNDFFYPLTLDYYREQKAVKQLHEVTNSVIESR<br>MKHLESSGGKVKENTDELGRKKKLAFLDILLQSTVDGKPLSIEDIREEVD<br>TFMFEGHDTTASAMTFTSFLLAGHPEVQARALEEQKSVFADDLLRPATY<br>KDLMEMKYLECVIKESLRIYPSVPLYGRHTHEDVNYKGNVIPKGTDITIF<br>DYGILHDPEIYPDPEKFDPSRFEEIDGKRPFNYIPFSAGPRNCIGQKFAMW<br>EMKCTLSKILRNFELRPAIPKHTLKLTPEAVLKSSNGVRIQVRPRKL
CYP4BN	WDSYKFLNNWLGKGLLTVEGSKWKKSRKLLTPAFHFSIVDQFVEVFDN<br>NANILVGLLEKEQESDFVDVFPYITMYTLDNICEATMGVSINAQTDTQSE<br>YVFAVKEMCRITVDRTFSPIKMFNTLYPLTADYYK
CYP4G29	MSAATASVDLENPTTLLTPKNIFYFLLIPALVLWYAYWKISRRHMVELAS<br>KIPGPEGLPLLGSALEFVGTSADIFKRMYAKSFEYGNTVKVWIGPKLLIFL<br>VDPRDVEIILSSHVHIDKASEYRFFQPWLGDGLLISTGQKWRAHRKLIAPT<br>FHLNVLKSFIDLFNANSREVVQKLKKEVGKEFDCHDYMSEATVEILLET<br>AMGVSKKTQDQSGYDYAMAVMKMCDILHLRHTKVWLRPDFIFNLTNY<br>AKKQEGLIGIIHSLTRKVIKRKRADFEKGIRGSTAEVPEELKTKNFDKNVS<br>SKTVVEGLSYGQAAGLKDDLDVDDDVGEKKRMAFLDLMIEASQNGVVI<br>NDEEIKEQVDTIMFEGHDTTAAGSSFFLSMMGVHQDIQDKVVQEIDEIFG<br>DSDRPATFADTLEMKYLERCLMETLRMYPPVPIIARQLRQDVKLASGDY<br>TLPAGATIVIGTFKIHRQEDVYPNPDKFDPDNFLPERSANRHYYSFIPFSA<br>GPRSCVGRKYAMLKLKILLSTILRNYRIYSTVEEKDFQLQGDIILKRADGF<br>RIKLEPRKRVLKA
CYP4G34	FSAGPRSCVGRKYAMLKLKVLLAGVLRKFQIHCSETEEDFKLQADIILKK<br>TGGFNISVTERKH
CYP4G[a]	WFVSKCETRMSEQVVISMEGESYSFVSSAITFILCLTAILTAYHYWFKNL<br>RHVKLTDNIPSPRTLPILGHIHMGIGKSPTDVYDLVYDLYHKLNSDIVKL<br>YFGPKLYIGICSAEDAELILGSSVHLEKPQEYRLFEPWLGDGLLISKGEKW<br>RSHRKMIAPTFHTSILKSFMPVFNKNANNLVEQF
CYP4G[b]	SKEKEDYMARKKSGETSLYMRAVKTGDYEDNQGSQDIMTNYIKDDLDE<br>NDDNDVGEKKRLAFLDFMIEASQTGNNITDEEIKEEVDTIMFEGHDTTAA<br>GSSFALCLLGIHQDIQQKVYEELKDIFQDNMDRPITFADTINMKYLERVIL<br>ETLRLYPPVPLIG
CYP4Q10	MMKVTIAMRSKKLALLDLLLNAKMNSGDIDDEGIRDEVNTFMFEGFDT<br>TSTSICFTLMVLANYKNWQELAYQEIIQVLGDSKKEPSIHDLNEMKILERI<br>IKESLRLYPSVPFIARILEEDTMVCSRVIPKGVPINIHIYDIHRDPKHWPDPE<br>KFDPDRFLTDNCVNRHPFAFVPFSAGPRNCIGQKFAMLELKTVYCGILRN<br>FILEPVD

CYP4Q11	GPRNCIGQKFAMLEIKAVLCGILRNFALEPQDTPDTIKIVPDLVLRTTDGKIRVKFRSRQ
CYP4Q[a]	PAETFTMFFFSVLVITAGCVTIYFLVKYLGEYLRCVEMMRKLPGPKTVPILGNTLDFVCDEVTIFLKIRNWCRTYGPVFRLNMSSFYPSVNIAGAEQFEVIASSMKNIRKPMVYSFLNRWLGEGLLTSTGSKWHTRRRILTPAFHFSILQQFVGIFNSETERLVQILKKETEHS
CYP4Q[b]	NVYSKFSVVVAGCVILFFLVRGFIEHLRTLKILKGLPGPEPIPVLKNVLEFLGDDITLFRKVRYWARTYGPIYRIDMTSLYPGVNITGAEEFEAIAGPMKHIQKAPIYFFLNRWLGEGLLTSTGSKWQTRRKNLDTSLSFQYFAAICWSFQ
CYP4Q[c]	PWTDVIPLISQFTLFTIAETSMGIKLNMENREHREYVESINKLGPLIFHRLLRPWLHNNFLYYHAFVNGFKERKLINTIHRFTDSIIAEKLKNFKKFEKGDGEYDFSQRKKLALLDLLLNAKLGEGIIDDEGIRDEVNTFMFEGYDTTSTAICFILMLLSIHKNWQDLVHEEILQVLGDSTEDPSLNDLNDLKIMERVVKECLRLYPSVPFIGRLLEEDTMINGFLIPKQTLMHVHILISIGI
CYP4[a]	RWFDSVYVRTMDSKVALEWSFLPPILFGIAITIFTYVWFSRREYLRKIEWVEEVPGLPFLGCILNLSKSTEILNVFSNYCRQYNGLARLNLFGKRFLLVSDYKFLEFVLNSNEILDKNDSYKF
CYP4[b]	RSLMMFLLWELFILVMVFLSFPVVYHYSKRNSKYLRNLPGPKPNMFVGNWIDFFGTTEEFLSTLMLYLEKYGPHIKVYNGPISTAVVSADEKFIEFFLSSPKLIDKADEYSFLHNWLGLGLLTSTGLKW
CYP4[c]	NPAVDMLLEICFGVAIACIITLSFSNYVVDCIRFSRLLSKLPGPKPLPVIGNILELACERELFFRKIRKWSQEYAPMYILSAFKSPAVNVSGAETFEVIA
CYP4[d]	TRIISYFPQREAMFSFMFFIVLCICLVSIFILYRGHTYLRKIPKPQCNFILGHIPDFIDRTKILEKLTKYSIECDGLMRIYFPPTKPAILIAKPSDVRTLLSSGAATKKSKFYDYMKVWLGDGLVTRDEGETWRTRRKLL
CYP4[e]	KRYAMLVLKNTISRIIKEFELLPVPDHKLELGMALVLKSKYGLPIRFKPRPKIEKDYCLMRKHSEKQINV
CYP349B	QVLPGEIAKRHPCSYVPFSYGPRNCIGPRYAMMAMKSLLCSVLRKYKIFTSYENVEDVKLKANSVLRPKDGYKVSVELRNI
CYP349C1	SIERLPFLNLMLKDPSFTMEEVIAEAKTFCLAGSETNASSTCFLLNLLGLYPEVQQKVYEEIIDILGPDRTILASDLPQMKYTERVIKENLRLFPVATIFGREIGEDIDLGNAILPKGSSVYFTAHYIHRNPKYWTNPLKFDPDRFLPDEIAKRHPCTFIPFLYGPRNCLGWKYALLNMKIIIA
CYP350D1	PFSAGPRNCIGWKYAIANMKTIIATVIRQFKIYTEYKSVEEIEINLYLLMRMRDGPKVWLENR
CYP350D2	IIREFKIFTEYKSIEEIEVKLYMLSKLKDRSKFWLELRQ
CYP350D4	VVREFKIFTEYKSIEEMEVVFHFTIQLKNGPKVWLERRR

CYP411?	CVKDVLRLFPIAPFMLRKVTEDYKLDKWILPKGATVAITAHYFHMNNAF<br>WENPKHFHPDHFLPEAVGKRHIYSYLPFSAGPRGCIGKTFANISIKTFLIHL<br>LQRFEIEADGKVPDIALMSDISVRPKKGFNVRLKKRVWS
CYP421A1	MIIALFSLVGVVLFPFLYWYYVVYVKFRSFRALPGPKPIPIIGSKIANDSVG<br>ILVDMQNLYERYKPTFVIYTGPQVRVVHSKPEHIHYIVTSNAHTTKSDNF<br>DVLKGWIGEGLATSKGDKWRERRKMINQSFNMNLMERFMQVFNSASD<br>ALVKNLEKESGKDCTDILNYTNFSALEIACENLMGIKLKPEENAAEEEYI<br>EKTKVMLRIVGVRFFSFWQRFETIFLMFSKHSKIYLDYVGTLKKFTLDVI<br>ERRSKTF

Table S3. Statistical analyses of five candidate reference genes based on their threshold cycle (C_T) value.

BestKeeper analyses of candidate reference genes (n=5)						
Parameter	*RpL4*	*Rp18*	*Ef1α*	*NADH*	*HSP70*	*BestKeeper*
N	72	72	72	72	72	72
GM [C_T]	17.10	16.85	13.41	14.12	14.91	15.21
AM [C_T]	17.12	16.87	13.43	14.14	14.93	15.22
Min [C_T]	15.20	15.05	11.48	12.65	13.04	13.51
Max [C_T]	19.07	18.86	15.54	15.51	17.38	17.19
SD [± C_T]	0.54	0.50	0.60	0.52	0.59	0.51
CV [% C_T]	3.15	2.96	4.45	3.67	3.98	3.33
Pair-wise correlation analyses						
BestKeeper vs	*RpL4*	*Rp18*	*Ef1α*	*NADH*	*HSP70*	
Coeff. of corr. [r]	0.973	0.965	0.968	0.811	0.960	
Coeff. of det. [r^2]	0.947	0.931	0.937	0.658	0.922	
P-value	0.001	0.001	0.001	0.001	0.001	

Abbreviations for listed parameters: n: number of candidate reference genes; N: sample size for each candidate reference genes as well as the *BestKeeper*; GM [C_T]: geometric means of the threshold cycle (C_T); AM [C_T]: the arithmetic mean of C_T; Min [C_T] and Max [C_T]: the extreme values of C_T; SD [± C_T]: the standard deviation of the C_T; CV [% C_T]: the coefficient of variance expressed as a percentage at the C_T level; The correlation between each candidate reference gene and *BestKeeper* index is calculated by the Pearson correlation coefficient [r], coefficient of determination [r^2], and the *P*-value.

Table S4. Differential expression of 76 CPB P450s in imidacloprid resistant and susceptible strains.

Primer No.	Gene Name	Fold[a)]	p-value	Differential expression[b)]
Ld85	CYP6BH	3.31	0.00	UP
Ld30	CYP6BJ1	1.34	0.01	
Ld113	CYP6BJ3	1.54	0.09	
Ld15	CYP6BJ[a]	1.97	0.00	
Ld84	CYP6BJ[b]	1.44	0.01	
Ld106	CYP6BK	1.62	0.01	
Ld154	CYP6BQ15	1.38	0.01	
Ld143	CYP6BQ16	1.99	0.00	
Ld27	CYP6BQ21	2.71	0.00	UP
Ld148	CYP6BQ22	1.48	0.03	
Ld161	CYP6BQ	1.96	0.03	
Ld45	CYP6BU[a]	1.51	0.05	
Ld157	CYP6BU[b]	1.20	0.07	
Ld2	CYP6ED1v2	1.46	0.02	
Ld104	CYP6ED	0.94	0.43	
Ld5	CYP6EE1v2	1.82	0.00	
Ld78	CYP6EE1	1.78	0.01	
Ld12	CYP6EF1	1.62	0.01	
Ld80	CYP6EG1	1.08	0.30	
Ld6	CYP6EH1	7.26	0.00	UP
Ld115	CYP6EZ1	7.20	0.00	UP
Ld93	CYP6FA1	8.29	0.01	UP
Ld101	CYP6	4.19	0.00	UP
Ld126	CYP9A	9.29	0.00	UP
Ld13	CYP9AV1	0.94	0.36	
Ld29	CYP9V1v1	3.22	0.00	UP
Ld25	CYP9V1v2	2.75	0.00	UP
Ld41	CYP9V2v2	2.70	0.00	UP
Ld83	CYP9Y[a]	15.19	0.00	UP
Ld114	CYP9Y[b]	7.45	0.00	UP
Ld117	CYP9Y[c]	10.13	0.00	UP
Ld42	CYP9Z13	2.63	0.00	UP
Ld63	CYP9Z14v1	7.25	0.00	UP
Ld38	CYP9Z25	16.64	0.00	UP
Ld3	CYP9Z26	1372.95	0.00	UP
Ld68	CYP9Z28	8.40	0.00	UP

Ld147	CYP9Z29	7.55	0.00	UP
Ld39	CYP9Z30	0.20	0.00	DOWN
Ld134	CYP9Z31	0.30	0.00	DOWN
Ld152	CYP9Z[a]	0.92	0.32	
Ld140	CYP9Z[b]	1.61	0.00	
Ld40	CYP9Z[c]	3.89	0.00	UP
Ld37	CYP9Z[d]	10.18	0.00	UP
Ld121	CYP9Z[e]	1.79	0.00	
Ld43	CYP9Z[f]	2.80	0.00	UP
Ld158	CYP9Z[g]	4.01	0.00	UP
Ld156	CYP9Z[h]	0.88	0.24	
Ld1	CYP9Z[i]	5.26	0.00	UP
Ld155	CYP9Z[j]	5.59	0.00	UP
Ld150	CYP9Z[k]	5.15	0.00	UP
Ld97	CYP9Z[l]	1.62	0.01	
Ld159	CYP9Z[m]	4.74	0.00	UP
Ld160	CYP9Z[n]	2.11	0.00	UP
Ld21	CYP9Z[o]	1.04	0.42	
Ld109	CYP9	1.54	0.04	
Ld131	CYP4AA1	4.83	0.01	UP
Ld50	CYP4BN12v2	1.38	0.11	
Ld118	CYP4BN12v3	1.36	0.11	
Ld48	CYP4BN13v4	6.20	0.00	UP
Ld49	CYP4BN14v1	0.51	0.00	
Ld89	CYP4BN15	4.26	0.01	UP
Ld153	CYP4BN	0.55	0.01	
Ld32	CYP4G29	3.27	0.00	UP
Ld127	CYP4G34	2.13	0.00	UP
Ld81	CYP4G[a]	2.46	0.00	UP
Ld141	CYP4G[b]	2.43	0.00	UP
Ld125	CYP4Q10	3.81	0.00	UP
Ld46	CYP4Q11	3.97	0.01	UP
Ld22	CYP4Q[a]	1.11	0.18	
Ld166	CYP4Q[b]	6.27	0.00	UP
Ld119	CYP4Q[c]	4.56	0.02	UP
Ld138	CYP4[a]	0.50	0.00	

Ld133	CYP4[b]	0.74	0.11	
Ld123	CYP4[c]	6.61	0.00	UP
Ld72	CYP4[d]	0.97	0.45	
Ld107	CYP4[e]	16.36	0.00	UP

a) Fold: the relative expression of P450 in the resistant strain divided by the expression in the susceptible strain; b) UP: Resistant/Susceptible > 2, p-value < 0.05; DOWN: Resistant/Susceptible < 0.5, p-value < 0.05.

Table S5. Induction of 76 CBP P450s by imidacloprid and potato leaf GAs in head, fat body and gut.

41 P450s were induced by both potato leaf GAs and imidacloprid in the resistant strain (fold>2, p-value<0.05)													
**Primer No.**	**Gene Name**	**Imidacloprid**						**Potato leaf GAs**					
		**Head**	**p-value**	**Fat body**	**p-value**	**Gut**	**p-value**	**Head**	**p-value**	**Fat body**	**p-value**	**Gut**	**p-value**
Ld85	CYP6BH	1.11	0.22	2.24	0.01	1.25	0.05	1.66	0.06	3.67	0.01	3.75	0.00
Ld30	CYP6BJ1	0.82	0.01	6.83	0.02	1.07	0.39	1.31	0.00	2.88	0.00	6.77	0.00
Ld15	CYP6BJ[a]	0.97	0.40	7.48	0.02	1.17	0.27	1.99	0.00	2.63	0.01	7.10	0.00
Ld84	CYP6BJ[b]	1.45	0.04	6.74	0.02	1.14	0.28	2.53	0.00	2.47	0.01	6.17	0.00
Ld154	CYP6BQ15	1.73	0.02	3.29	0.00	1.60	0.00	1.11	0.32	1.30	0.10	5.15	0.00
Ld143	CYP6BQ16	0.57	0.11	5.48	0.00	1.39	0.05	0.82	0.29	2.76	0.00	4.30	0.01
Ld27	CYP6BQ21	1.63	0.01	6.87	0.00	1.51	0.16	2.13	0.00	3.36	0.00	5.60	0.04
Ld148	CYP6BQ22	0.93	0.43	4.67	0.00	1.55	0.01	1.25	0.27	1.90	0.01	5.13	0.01
Ld161	CYP6BQ	0.97	0.43	5.10	0.00	1.78	0.00	1.40	0.04	2.45	0.00	4.94	0.00
Ld45	CYP6BU[a]	0.79	0.25	4.63	0.00	1.52	0.00	1.27	0.26	2.01	0.01	4.92	0.00
Ld157	CYP6BU[b]	1.17	0.21	4.79	0.00	1.69	0.02	2.04	0.01	2.09	0.00	6.23	0.01
Ld2	CYP6ED1v2	1.07	0.25	1.83	0.03	3.13	0.01	1.69	0.04	2.92	0.01	16.35	0.03
Ld12	CYP6EF1	0.81	0.08	2.44	0.00	1.31	0.02	0.87	0.21	1.99	0.00	3.22	0.00
Ld115	CYP6EZ1	1.40	0.01	2.01	0.04	1.42	0.05	0.45	0.00	1.69	0.02	4.70	0.04
Ld126	CYP9A	2.12	0.00	3.76	0.01	1.98	0.01	2.28	0.00	2.40	0.00	2.78	0.00
Ld29	CYP9V1v1	1.74	0.00	2.89	0.00	3.63	0.03	0.83	0.01	0.88	0.32	4.45	0.00
Ld25	CYP9V1v2	1.64	0.03	2.98	0.00	3.79	0.04	0.75	0.01	1.04	0.44	4.32	0.00
Ld41	CYP9V2v2	1.64	0.03	3.14	0.00	3.69	0.02	0.87	0.03	1.06	0.41	4.95	0.00
Ld83	CYP9Y[a]	2.55	0.00	4.00	0.01	2.05	0.00	3.50	0.00	2.51	0.00	3.12	0.00
Ld114	CYP9Y[b]	3.42	0.00	3.80	0.03	1.78	0.00	3.56	0.00	2.72	0.02	3.42	0.00
Ld117	CYP9Y[c]	2.46	0.00	2.19	0.00	2.50	0.00	2.40	0.00	1.88	0.00	1.28	0.01
Ld38	CYP9Z25	3.35	0.00	7.74	0.00	5.40	0.00	4.26	0.00	11.44	0.00	21.41	0.03
Ld3	CYP9Z26	4.72	0.00	24.29	0.00	6.34	0.00	5.76	0.00	10.73	0.00	10.09	0.00

Ld147	CYP9Z29	1.89	0.00	9.79	0.00	1.83	0.00	2.34	0.00	7.35	0.00	8.51	0.00
Ld152	CYP9Z[a]	0.23	0.07	5.19	0.00	0.84	0.10	0.31	0.09	3.90	0.00	5.35	0.00
Ld140	CYP9Z[b]	1.39	0.06	4.01	0.00	3.01	0.00	1.90	0.04	1.94	0.02	3.71	0.00
Ld40	CYP9Z[c]	2.31	0.00	4.70	0.01	3.13	0.00	1.61	0.12	0.71	0.07	7.53	0.00
Ld37	CYP9Z[d]	2.73	0.00	13.48	0.00	3.53	0.00	3.51	0.00	5.33	0.00	6.00	0.00
Ld121	CYP9Z[f]	0.81	0.12	2.22	0.00	1.43	0.05	1.06	0.01	1.58	0.16	2.65	0.00
Ld43	CYP9Z[e]	0.76	0.25	2.82	0.02	1.96	0.01	1.32	0.23	0.64	0.21	3.10	0.00
Ld158	CYP9Z[g]	1.98	0.00	9.25	0.00	1.80	0.00	1.55	0.02	4.50	0.00	7.48	0.00
Ld150	CYP9Z[k]	2.09	0.00	4.04	0.00	2.60	0.00	1.23	0.05	0.75	0.22	7.12	0.00
Ld97	CYP9Z[l]	1.81	0.02	4.35	0.00	3.41	0.00	1.91	0.00	2.78	0.00	4.80	0.00
Ld159	CYP9Z[m]	1.84	0.00	4.79	0.00	2.67	0.00	1.10	0.23	0.91	0.48	7.85	0.00
Ld160	CYP9Z[n]	1.13	0.13	2.27	0.00	1.51	0.02	1.19	0.10	1.48	0.15	2.16	0.00
Ld109	CYP9	1.01	0.49	1.83	0.04	2.33	0.01	1.57	0.03	2.55	0.01	11.99	0.04
Ld50	CYP4BN12v2	1.39	0.05	2.19	0.00	1.65	0.05	2.01	0.00	3.59	0.00	6.82	0.01
Ld32	CYP4G29	0.90	0.00	2.13	0.02	2.52	0.01	1.09	0.38	2.16	0.02	1.50	0.18
Ld22	CYP4Q[a]	0.80	0.01	0.78	0.04	3.00	0.00	0.54	0.00	0.47	0.00	3.61	0.03
Ld166	CYP4Q[b]	1.31	0.20	2.30	0.00	1.37	0.00	0.84	0.13	2.22	0.03	2.85	0.00
Ld133	CYP4[b]	0.81	0.33	2.30	0.01	2.05	0.05	1.06	0.44	3.24	0.02	7.56	0.01
**13 P450s were induced by imidacloprid alone in the resistant strain (fold>2, p-value<0.05)**													
**Primer No.**	**Gene Name**	**Imidacloprid**						**Potato leaf GAs**					
		**Head**	**p-value**	**Fat body**	**p-value**	**Gut**	**p-value**	**Head**	**p-value**	**Fat body**	**p-value**	**Gut**	**p-value**
Ld13	CYP9AV1	0.39	0.06	4.59	0.01	0.29	0.11	0.75	0.29	1.11	0.29	0.02	0.04
Ld42	CYP9Z13	1.38	0.08	2.53	0.00	0.61	0.00	1.15	0.07	1.42	0.10	2.30	0.39
Ld63	CYP9Z14v1	1.40	0.02	4.79	0.00	2.11	0.00	1.31	0.00	1.72	0.02	1.18	0.02
Ld68	CYP9Z28	1.48	0.02	4.82	0.00	4.82	0.00	1.32	0.03	1.60	0.02	1.60	0.02
Ld156	CYP9Z[h]	0.38	0.06	4.47	0.00	0.33	0.12	0.61	0.19	1.10	0.29	0.02	0.04
Ld1	CYP9Z[i]	1.49	0.00	2.49	0.00	1.96	0.00	1.56	0.02	1.06	0.40	1.01	0.48

Ld155	CYP9Z[j]	1.19	0.02	2.23	0.00	1.93	0.00	0.96	0.27	0.83	0.08	0.96	0.39
Ld21	CYP9Z[o]	0.62	0.14	3.78	0.00	0.33	0.11	1.20	0.30	0.85	0.20	0.03	0.04
Ld131	CYP4AA1	1.24	0.05	2.67	0.00	0.01	0.00	1.81	0.00	0.78	0.00	0.01	0.00
Ld127	CYP4G34	0.92	0.17	2.38	0.02	2.12	0.01	0.83	0.17	1.79	0.07	0.38	0.00
Ld81	CYP4G[a]	1.10	0.13	2.38	0.02	3.24	0.01	0.95	0.41	1.59	0.11	0.59	0.18
Ld141	CYP4G[b]	0.87	0.03	2.35	0.02	1.66	0.04	0.79	0.10	1.70	0.08	0.34	0.00
Ld125	CYP4Q10	0.96	0.42	2.64	0.00	1.22	0.00	0.63	0.05	1.25	0.00	1.80	0.00
**17 P450s were induced by potato leaf GAs alone in the resistant strain (fold>2, p-value<0.05)**													
**Primer No.**	**Gene Name**	**Imidacloprid**						**Potato leaf GAs**					
		**Head**	**p-value**	**Fat body**	**p-value**	**Gut**	**p-value**	**Head**	**p-value**	**Fat body**	**p-value**	**Gut**	**p-value**
Ld113	CYP6BJ3	1.29	0.17	1.68	0.02	1.20	0.14	0.87	0.10	2.17	0.00	7.47	0.00
Ld106	CYP6BK	0.27	0.09	1.96	0.00	1.49	0.00	0.23	0.08	0.64	0.03	2.05	0.00
Ld104	CYP6ED	3.25	0.07	6.54	0.06	8.23	0.05	5.74	0.01	7.90	0.00	5.10	0.03
Ld78	CYP6EE1	0.87	0.26	1.83	0.04	1.20	0.17	0.92	0.28	2.99	0.07	2.06	0.00
Ld93	CYP6FA1	0.76	0.25	0.34	0.12	2.17	0.06	1.32	0.01	0.32	0.11	4.69	0.00
Ld101	CYP6	0.92	0.38	0.29	0.10	1.96	0.02	1.33	0.05	0.38	0.13	4.60	0.00
Ld118	CYP4BN12v3	1.90	0.00	1.61	0.03	1.94	0.04	1.73	0.00	2.36	0.01	7.03	0.01
Ld48	CYP4BN13v4	0.57	0.02	0.92	0.42	0.76	0.23	1.27	0.07	1.29	0.34	3.28	0.00
Ld49	CYP4BN14v1	0.92	0.00	0.36	0.00	1.67	0.00	1.33	0.03	0.63	0.00	2.76	0.00
Ld89	CYP4BN15	0.75	0.10	1.26	0.10	1.80	0.01	0.80	0.13	1.35	0.06	4.99	0.00
Ld153	CYP4BN	1.32	0.01	0.37	0.00	1.52	0.00	0.82	0.05	0.35	0.01	3.57	0.00
Ld46	CYP4Q11	0.77	0.17	1.88	0.00	1.28	0.00	0.63	0.06	1.28	0.02	4.24	0.00
Ld119	CYP4Q[c]	0.99	0.48	1.55	0.01	1.23	0.02	0.52	0.03	1.16	0.05	3.85	0.00
Ld138	CYP4[a]	0.98	0.47	0.36	0.00	1.50	0.00	0.67	0.14	0.33	0.02	3.44	0.00
Ld123	CYP4[c]	0.59	0.12	1.52	0.00	1.13	0.06	0.51	0.05	1.21	0.23	4.08	0.00
Ld72	CYP4[d]	0.33	0.07	0.75	0.17	1.12	0.25	0.50	0.14	0.61	0.09	4.50	0.00
Ld107	CYP4[e]	0.77	0.21	1.43	0.19	1.55	0.01	6.27	0.03	8.88	0.00	9.71	0.00

5 P450s were not induced by imidacloprid or potato leaf GAs in the resistant strain													
**Primer No.**	**Gene Name**	**Imidacloprid**						**Potato leaf GAs**					
		**Head**	**p-value**	**Fat body**	**p-value**	**Gut**	**p-value**	**Head**	**p-value**	**Fat body**	**p-value**	**Gut**	**p-value**
Ld5	CYP6EE1v2	0.80	0.18	1.56	0.03	0.98	0.47	1.06	0.40	1.44	0.05	1.94	0.00
Ld80	CYP6EG1	0.18	0.07	1.32	0.29	1.30	0.00	0.10	0.06	0.21	0.02	1.83	0.00
Ld6	CYP6EH1	0.89	0.24	1.94	0.06	1.63	0.00	0.39	0.00	1.51	0.07	4.34	0.05
Ld39	CYP9Z30	0.61	0.00	0.70	0.09	1.04	0.45	1.02	0.44	0.76	0.17	1.62	0.01
Ld134	CYP9Z31	0.65	0.00	0.73	0.12	0.96	0.44	0.97	0.44	0.64	0.11	1.52	0.05

Table S6. Insect P450s selected for phylogenetic tree construction.

Insect species	P450 name	Access number	Length (aa)	Reference
Apis mellifera	CYP9Q1	---	510	Mao et al. 2011[66]
Apis mellifera	CYP9Q2	---	532	Mao et al. 2011[66]
Apis mellifera	CYP9Q3	---	517	Mao et al. 2011[66]
Bemisia tabaci	CYP6CM1vQ	ACA51846	521	Karunker et al. 2008[33]
Bemisia tabaci	CYP6CM1vB	ACD84797	520	Karunker et al. 2008[33]
Drosophila melanogaster	CYP6g1	AAL89788	524	Daborn et al. 2002[29]; Hoi et al. 2014[30]
Helicoverpa armigera	CYP6AE11	AID54887	522	Tao et al. 2012[14]
Helicoverpa armigera	CYP6AE14	AKS48888	526	Tao et al. 2012[14]
Helicoverpa armigera	CYP6B7	AKS48890	504	Tao et al. 2012[14]
Helicoverpa armigera	CYP9A14	AKS48891	530	Tao et al. 2012[14]
Helicoverpa armigera	CYP9A17	AAV28704	531	Zhou et al. 2010[45]
Helicoverpa armigera	CYP321A1	AAM54724	499	Tao et al. 2012[14]
Meligethes aeneus	CYP6BQ23	AGQ51764	522	Zimmer et al. 2014[32]
Musca domestica	CYP4D4v2	ABV48807	505	Zhu et al. 2008a[21]
Musca domestica	CYP4G2v1	ABV48808	549	Zhu et al. 2008a[21]
Musca domestica	CYP6A5v2	ABV48810	507	Zhu & Liu 2008[39]
Musca domestica	CYP6A38v1	ABV48809	500	Zhu et al. 2008a[21]
Musca domestica	CYP6A36	ABG34551	507	Zhu et al. 2008b[38]
Musca domestica	CYP6D1	AAA81513	516	Tomita et al. 1995[28]
Myzus persicae	CYP6CY3-S	KF218356	511	Puinean et al. 2010[34]
Myzus persicae	CYP6CY3-R	KF218350	511	Puinean et al. 2010[34]
Manduca sexta	CYP9A4	AAD51036	522	Stevens et al. 2000[43]
Manduca sexta	CYP9A5	AAD51038	520	Stevens et al. 2000[43]
Manduca sexta	CYP4M1	ADE05575	504	Snyder et al. 1995[25]; Pauchet et al. 2010[44]
Manduca sexta	CYP4CG1	ADE05577	500	Snyder et al. 1995[25]; Pauchet et al. 2010[44]
Nilaparvata lugens	CYP6AY1	CAH65682	501	Bao et al. 2015[37]; Ding et al. 2013[36]
Nilaparvata lugens	CYP6ER1	AEK01112	506	Bao et al. 2015[37]; Bass et al. 2011[35]
Papilio polyxenes	CYP6B1v1	AAA29789	498	Petersen et al. 2001[27]
Papilio polyxenes	CYP6B3	AAA96255	498	Petersen et al. 2001[27]
Tribolium castaneum	CYP6BQ9	NP_001177722	521	Zhu et al. 2010[26]; 2013[31]

Table S7. Primers used for qRT-PCR.

Primer No.	Gene Name	Primer name	Sequence (5' to 3')
Ld85	*CYP6BH*	qContig04303F	GGTCACTCGAAGTCTTGTATACACCC
		qContig04303R	ACGAAGATGGGATGCAAGATCGGA
Ld30	*CYP6BJ1*	qLdDQ117463F	ACAGGCGATGGAACTAGCTTGAC
		qLdDQ117463R	TTGAACTCGTTTCGAATCCGGC
Ld113	*CYP6BJ3*	qContig12558F	TGAGACCATAGCAAACTGCGGACT
		qContig12558R	TTACAGTCCAAGCCGAAAGCACAG
Ld15	*CYP6BJ*[a]	qLdEB757598F	ACCGGTGGATTTGGAACTAATCA
		qLdEB757598R	CAGGTTCCTCCATTTCTTTCCTCC
Ld84	*CYP6BJ*[b]	qContig10557F	GGTTGAGATTCGGTGTGATGCAGT
		qContig10557R	TTCAGCCACATCCCTCCTTCTGTT
Ld106	*CYP6BK*	qContig07056F	TTGGAGATTACATCCACCAGCCCA
		qContig07056R	TGGATCGAACACCAGTGGATTAGG
Ld154	*CYP6BQ15*	qContig08488F	AGTCAAAGTGGGTCTCACTGCCA
		qContig08488R	AGTCAAAGTGGGTCTCACTGCCA
Ld143	*CYP6BQ16*	qContig01265F	TACTCGGCCATTCAAGATGCAGTG
		qContig01265R	AAAGCAACTGAACCAATCGCGTCG
Ld27	*CYP6BQ21*	qContig00220F	GGGTGTGGAATACATCGAACCTGA
		qContig00220R	GGTCAATAGGAATGATAACTGGCG
Ld148	*CYP6BQ22*	qContig01264F	TCACACCGACCTTCACATCAGGAA
		qContig01264R	ATTGAACCAATCGCGTCTGTTGCG
Ld161	*CYP6BQ*	qContig10876F	AGACTCTTCGCAAACATTCAGCCG
		qContig10876R	CGATGGATCCCTGAAACGGGAATA
Ld45	*CYP6BU*[a]	qContig09262F	TGCATAGCGGCCAGATATGGACTT
		qContig09262R	ATCGAGCCAAACTCCTCCCTCAAT
Ld157	*CYP6BU*[b]	qContig09308F	ACCTAGAATGTGCATAGGAGCCAG
		qContig09308R	TTACGTTGAGCCAAACTCCTCCCT
Ld2	*CYP6ED1v2*	qContig01354F	TCAAACTCATCTCTTCTGTTGGTATAGTC
		qContig01354R	CTCTGTAGTGATCCGTGAATTCCAA
Ld104	*CYP6ED*	qContig10723F	CCGAGGAATTGTGTAGGTAACCGT
		qContig10723R	CGGCAATATCGTTGAGGAACTCAC
Ld5	*CYP6EE1v2*	qContig01835F	TCTCATGGTGCTACGGTCATTGGA
		qContig01835R	ATTCCAAGTGTGGACCTGGAAACC
Ld78	*CYP6EE1*	qContig03888F	CCAAGTTTGGAAAGGGAATGCGTG
		qContig03888R	ATCCAAACGGTGGTTCCCTTCTCA
Ld12	*CYP6EF1*	qLdEB757657F	TCTCGGCACCTTCTCAATGGTGAT
		qLdEB757657R	TCCTGAACAAGGATGAGCTGTTGC
Ld80	*CYP6EG1*	qContig02306F	TACATTCACAGGCTGAGAGCCGTT
		qContig02306R	AGCGCTCACCTCTTATCCCTTGAT
Ld6	*CYP6EH1*	qContig04994F	TCAACATGTTCGACCTATCGCCGT
		qContig04994R	ACCTCTCCGAAGCTTATCTTTCCG
Ld115	*CYP6EZ1*	qContig01667F	CCCGGAACAATTCAATCCTGAGAG
		qContig01667R	TAGGCCCAATTTGGTTTGCCACAG
Ld93	*CYP6FA1*	qContig00754F	ACGCTTCGGAATGGTACAGGTCAA
		qContig00754R	CCAAATACCTCCTTTAGCGCTTGC
Ld101	*CYP6*	qContig07881F	AAATGGATGGGATGTGGCAAACGG
		qContig07881R	ATCCTCCTCCGTGTCTCAAACCTT
Ld126	*CYP9A*	qContig09946F	CTACTTGTGCCTTTGGCGTGCAAT
		qContig09946R	GTTGGCTTCGTCATTCTTGAACCC
Ld13	*CYP9AV1*	qContig00465F	AAATGACCTCGCCAGGTTTGATGG
		qContig00465R	AAGAGCGGATCAAGGTCATCGTGT
Ld29	*CYP9V1v1*	qLdDQ117460F	TCCGACCTTGATGATAAGAGACCC
		qLdDQ117460R	TCTCTCCACCTTTGACCTGTCAGT

Ld25	*CYP9V1v2*	qLdEB754758F	ACCATAGGGTCGTCATGCCTGAAA
		qLdEB754758R	TGCCTGTGAATGCTGGACTAAGGA
Ld41	*CYP9V2v2*	qLdDQ631662F	CCCGGAATTGTATTGGTTCAAGATTTGCC
		qLdDQ631662R	CGAATTGAGGTTGAGCGTTGTCGT
Ld83	*CYP9Y*[a]	qContig11158F	TGGACCAAAGAGTACCCGAAGGAA
		qContig11158R	TCGACAATTCAGTCCGAGGTACGA
Ld114	*CYP9Y*[b]	qContig12257F	ACGTTACAAAGCTAGGAGACGGCA
		qContig12257R	CGGCCACACATATTCTGGTTAGCA
Ld117	*CYP9Y*[c]	qContig09375F	ATTTGGAAGTGGACCACGCAATTG
		qContig09375R	GCCAAATTCCTTCTCGTCCAGCAAG
Ld42	*CYP9Z13*	qLdDQ631659F	GGAGGGCCCAAGGAACTGTATT
		qLdDQ631659R	TCCTAACCAGAAACCACCTTCAGC
Ld63	*CYP9Z14v1*	qContig01918F	CACGGAATTGCATAGCGTCGAGAT
		qContig01918R	AATCCAAGCCAGAAGCCTCCTTCA
Ld38	*CYP9Z25*	qContig00260F	ACTTGCACACCTTTGAGAGCATCC
		qContig00260R	TTGTCAACTTTCTCATGCCCACCC
Ld3	*CYP9Z26*	qContig00493F	AGTTCTGGTGGCTGCTGTATTAGC
		qContig00493R	GTTCTCCAGTTCTCTCCAAATAGCC
Ld68	*CYP9Z28*	qContig01161F	ACCAATGTCTTACTGGAGAGAACGGG
		qContig01161R	ACTCCATAATACCTGGCACCTGGA
Ld147	*CYP9Z29*	qContig01238F	GAGCTGCTGCATTAGCCACCATTT
		qContig01238R	TCTTGTTTGACACCTCTCTCTCCC
Ld39	*CYP9Z30*	qContig03170F	CGGCTCCAGATTTGCTTTGTTGGA
		qContig03170R	CACCTTCAACGGAAACAAGGATCG
Ld134	*CYP9Z31*	qContig01532F	AAAGCTGGACTCAATGTGGATCGC
		qContig01532R	AGACATGGACCCATTGTGGGCTAA
Ld152	*CYP9Z*[a]	qContig06948F	GGCACATTATTGATGATCCCTATGGTGGG
		qContig06948R	TATACAGAGTCTTGGTCCCAGTCCG
Ld140	*CYP9Z*[b]	qContig03035F	TACCCAGAACCACTGCGTTTCGAT
		qContig03035R	GGTCCCGCACCAAATGGCATATAA
Ld40	*CYP9Z*[c]	qContig08090F	AGGGACCGAGGATATGTATAGGGT
		qContig08090R	CCTCTGCCGTTAGATTGAACGTCTT
Ld37	*CYP9Z*[d]	qContig04061F	TCAAATTCCACTGAAACTGTCTAGG
		qContig04061R	CCACTCCACATCCTGAATAACTACTG
Ld121	*CYP9Z*[e]	qContig08039F	TGCCATTGTTCCAGTCGAGA
		qContig08039R	CGCTTCAATCCCAACCAGAAACCA
Ld43	*CYP9Z*[f]	qLdDQ631658F	CGATCCAGAAAGATTCAGCGACGA
		qLdDQ631658R	GCCAAATCGTGATCCGATGCAGTT
Ld158	*CYP9Z*[g]	qContig10068F	GTTGGCTGTCCATCCAGACATTCA
		qContig10068R	TTACTTCTCCTCCACATTCCGCCA
Ld156	*CYP9Z*[h]	qContig09087F	AAACGAAAGTGCCTCTGGAAGTC
		qContig09087R	GTCTAAACGTTTCAGTCCTACCCA
Ld1	*CYP9Z*[i]	qContig06620F	ACGAGCCCATCATTTCTCAAGACG
		qContig06620R	GCTCTAGATTCGCTCTCTTGGAAGCA
Ld155	*CYP9Z*[j]	qContig08801F	ACTGGTTTGGTATTTGCGGCAGAC
		qContig08801R	TGGAACGTTGAGTTGACAAACGCC
Ld150	*CYP9Z*[k]	qContig06091F	AAGCTACTGGAGAGAACGAGGTG
		qContig06091R	GGTTGACCGGATTCATAGATCCCA
Ld97	*CYP9Z*[l]	qContig08836F	CCAGTAGTTCAACGGCTTAACGCA
		qContig08836R	TGATCAGTTGGCAGTGTCGTCGAT
Ld159	*CYP9Z*[m]	qContig10701F	TACCTCTGCCTTTGGTGTGGAAGT
		qContig10701R	TCCTCCCAAATCAGTTGCCTCCT
Ld160	*CYP9Z*[n]	qContig10810F	TGGGAAAGAAGGCCACAGATCTC
		qContig10810R	ACACCCAGAACCTTGCTGATCCA
Ld21	*CYP9Z*[o]	qLdEB755804F	AATGACCTCGCCAGGTTTGATGGT
		qLdEB755804R	TTGGTAAGAGCGGGTCAAGGTCAT

Ld109	*CYP9*	qContig11061F<br>qContig11061R	GCGCAGGTACCTTCCATTTGGTAT<br>AAACATGTTGATGGGCTCACAGCG
Ld131	*CYP4AA1*	qContig07817F<br>qContig07817R	TCGTGGGCTTTATTACCCAGATGC<br>TTGTTGGCTTCCTCAGCAGTTGTG
Ld50	*CYP4BN12v2*	qLdDQ631646F<br>qLdDQ631646R	CATCATCTCGAAAGTGCTGAGGCA<br>TGATCTCAGGAGCGAGCTTCAACT
Ld118	*CYP4BN12v3*	qContig01477F<br>qContig01477R	TTGCAAGTCATCGGAGTCCCAACT<br>AATTCCTTGGTCCTGCGCTAAAGG
Ld48	*CYP4BN13v4*	qLdDQ631649F<br>qLdDQ631649R	GCATTGGTTGGTAATGAGGAGGCA<br>TGGAGTCGCTGGACGTAGTTCAAA
Ld49	*CYP4BN14v1*	qContig01451F<br>qContig01451R	GACCACGAAACTGCATAGGCCAAA<br>CTAAGATTGGCACATGACCGCAAG
Ld89	*CYP4BN15*	qContig00384F<br>qContig00384R	AATGTCATGGCGGAAGCGGTAGTA<br>CATTTGGAAAGTTCGGGCGGGAAA
Ld153	*CYP4BN*	qContig07821F<br>qContig07821R	CGTGTCCATAAACGCTCAAACGGAC<br>TCTGTCGACCGTGATTCTGCACAT
Ld32	*CYP4G29*	qLdDQ117464F<br>qLdDQ117464R	TGGTACCTTCAAGATTCACCGCC<br>TGTTGGCACTACGTTCCGGTAAGA
Ld127	*CYP4G34*	qContig09239F<br>qContig09239R	TGCTAGCAGGCGTTCTCCGTAAAT<br>GCTTCAATGCTTCCTCTCAGTAACAG
Ld81	*CYP4G*[a]	qContig02645F<br>qContig02645R	ATGGGCATCGGAAAGTCACCTACA<br>CATCTTCCGCACTGCAGATTCCAA
Ld141	*CYP4G*[b]	qContig05926F<br>qContig05926R	TTCCAGGACAACATGGACAGACCT<br>ACTGGTGGATAGAGCCTCAGTGTT
Ld125	*CYP4Q10*	qContig04050F<br>qContig04050R	CATACCCAAGGGCGTACCGATC<br>ATGCGAACGGATGCCTGTTGAC
Ld46	*CYP4Q11*	qContig09919F<br>qContig09919R	GCTGGAGATTAAGGCAGTACTCTGTG<br>TTTGCCATCTGTCGTCCTCAGT
Ld22	*CYP4Q*[a]	qLdEB755786F<br>qLdEB755786R	TGTCGAGCTTCTACCCTTCAGTCA<br>CTGAAATGGAAGGCTGGCGTCAAA
Ld166	*CYP4Q*[b]	qContig02266F<br>qContig02266R	GACAGTGTTCACAACGCGTAGTAG<br>ATCACACATCCTGCCACCACAACTG
Ld119	*CYP4Q*[c]	qContig03154F<br>qContig03154R	ATACCACCTCTACGGCCATTTGCT<br>GGGTCTTCTGTTGAATCTCCAAGG
Ld138	*CYP4*[a]	qContig04089F<br>qContig04089R	ATTGAATGGGTTGAGGAAGTGCCC<br>TCAACCTGGCCAGTCCGTTGTATT
Ld133	*CYP4*[b]	qContig06673F<br>qContig06673R	GCAATCCCAATCCAAGCCAGTTGT<br>ACCAATCAGCACGGCAGTAGTTTC
Ld123	*CYP4*[c]	qContig09902F<br>qContig09902R	TTCAGCTCCTGATACATTGACGGC<br>CTCGCGTGTGAAAGGGAGTTGTTT
Ld72	*CYP4*[d]	qLdEB760335F<br>qLdEB760335R	CCATCGAATGTGATGGACTAATGAGG<br>TCGTCTCTTGTTACGAGTCCGTCT
Ld107	*CYP4*[e]	qContig10575F<br>qContig10575R<br>qContig02661R	CCAGTACCCGATCACAAACTGGAA<br>CGGCCTAGGTTTGAATCTTATTGGC<br>ACGAACGTTGGTTTATACCGCTCG
Ribosomal protein L4		qLdEB761170*F<br>qLdEB761170R	AAAGAAACGAGCATTGCCCTTCCG<br>TTGTCGCTGACACTGTAGGGTTGA
Ribosomal protein L18		qLdEB761179F<br>qLdEB761179R	TAGAATCCTCAAAGCAGGTGGCGA<br>AGCTGGACCAAAGTGTTTCACTGC
Elongation factor1α		qLdEB754313F<br>qLdEB754313R	AAGGTTCCTTCAAGTATGCGTGGG<br>GCACAATCAGCTTGCGATGTACCA
NADH dehydrogenase		qLdEB761955F<br>qLdEB761955R	TAGGATGGGACGGTTTGGGATTAG<br>GCAACATCGCCAATTCGGTTCCTT
Heat shock protein 70		qLdEB761546F<br>qLdEB761546R	ACCTCGATGGTCCTCACCAAGATG<br>GGGACAGTGATGTCTGCGTTCG

*This EB number stands for the access number in NCBI database.
